# Overcoming Indecision by Changing the Decision Boundary

**DOI:** 10.1037/xge0000286

**Published:** 2017-04-13

**Authors:** Gaurav Malhotra, David S. Leslie, Casimir J. H. Ludwig, Rafal Bogacz

**Affiliations:** 1 School of Experimental Psychology, University of Bristol; 2 Department of Mathematics and Statistics, Lancaster University; 3 School of Experimental Psychology, University of Bristol; 4 MRC Brain Network Dynamics Unit, University of Oxford

**Keywords:** decision making, decision threshold, decreasing bounds, optimal decisions, reward rate

## Abstract

The dominant theoretical framework for decision making asserts that people make decisions by integrating noisy evidence to a threshold. It has recently been shown that in many ecologically realistic situations, decreasing the decision boundary maximizes the reward available from decisions. However, empirical support for decreasing boundaries in humans is scant. To investigate this problem, we used an ideal observer model to identify the conditions under which participants should change their decision boundaries with time to maximize reward rate. We conducted 6 expanded-judgment experiments that precisely matched the assumptions of this theoretical model. In this paradigm, participants could sample noisy, binary evidence presented sequentially. Blocks of trials were fixed in duration, and each trial was an independent reward opportunity. Participants therefore had to trade off speed (getting as many rewards as possible) against accuracy (sampling more evidence). Having access to the actual evidence samples experienced by participants enabled us to infer the slope of the decision boundary. We found that participants indeed modulated the slope of the decision boundary in the direction predicted by the ideal observer model, although we also observed systematic deviations from optimality. Participants using suboptimal boundaries do so in a robust manner, so that any error in their boundary setting is relatively inexpensive. The use of a normative model provides insight into what variable(s) human decision makers are trying to optimize. Furthermore, this normative model allowed us to choose diagnostic experiments and in doing so we present clear evidence for time-varying boundaries.

In an early theory of decision making, Cartwright and Festinger (1943) modeled decision making as a struggle between fluctuating forces. At each instant, the decision maker drew a sample from the (Gaussian) distribution for each force and computed the difference between these samples. This difference was the resultant force and no decision was made while the opposing forces were balanced and the resultant force was zero. Cartwright and Festinger realized that if a decision was made as soon as there was the slightest imbalance in forces, there would be no advantage to making decisions more slowly. This was inconsistent with the observation that the speed of making decisions traded-off with their accuracy, a property of decision making that had already been recorded (Festinger, 1943; Garrett, 1922; Johnson, 1939) and has been repeatedly observed since (e.g., Bogacz, Wagenmakers, Forstmann, & Nieuwenhuis, 2010; Howell & Kreidler, 1963; Pachella, 1974; Wickelgren, 1977; Luce, 1986). Cartwright and Festinger addressed the speed–accuracy trade-off by introducing an internal *restraining force—*also normally distributed and in the opposite direction to the resultant force—which would prevent the decision maker from going off “half-cocked” (Cartwright & Festinger, 1943, p. 598). The decision maker drew samples from this restraining force and did not make a decision until the resultant force was larger than these samples. The restraining force was adaptable and could be adjusted on the basis of whether the decision maker wanted to emphasize speed or accuracy in the task.

In the ensuing decades, Cartwright and Festinger’s theory fell out of favor because several shortcomings (see Irwin, Smith, & Mayfield, 1956; Vickers, Nettelbeck, & Willson, 1972) and was superseded by the signal detection theory (Tanner & Swets, 1954) and sequential sampling models (LaBerge, 1962; Laming, 1968; Link & Heath, 1975; Ratcliff, 1978; Stone, 1960; Vickers, 1970). These models do not mention a restraining force explicitly, but this concept is implicit in a threshold, which must be crossed before the decision maker indicates their choice. Just as the restraining force could be adjusted based on the emphasis on speed or accuracy, these models proposed that the threshold could be lowered or raised to emphasize speed or accuracy. This adaptability of thresholds has been a key strength of these models, a feature that has been used to explain how distribution of response latencies changes when subjects are instructed to emphasize speed or accuracy in a decision (for a review, see Bogacz, Brown, Moehlis, Holmes, & Cohen, 2006; Ratcliff & Smith, 2004; Ratcliff, Smith, Brown, & McKoon, 2016).

Introducing a restraining force or a threshold to explain the speed–accuracy trade-off answers one question but raises another: How should a decision maker select the restraining force (threshold) for a decision-making problem? Should this restraining force remain constant during a decision? This problem was examined by Wald (1947) who proposed that, for an isolated decision, an optimal decision maker can distinguish between two hypotheses by choosing the desired ratio of Type I and Type II errors and then using a statistical procedure called the sequential-probability-ratio-test (SPRT). In the SPRT, the decision maker sequentially computes the ratio of the likelihoods of all observations given the hypotheses and the decision process terminates only once the ratio exceeds a threshold (corresponding to accepting the first hypothesis) or decreases below another threshold (corresponding to accepting the second hypothesis). The values of these thresholds do not change as more samples are accumulated and they determine the accuracy of decisions. Wald and Wolfowitz (1948) showed that the SPRT requires a smaller or equal number of observations, on average, than any other statistical procedure for a given accuracy of decisions.

The SPRT gives a statistically optimal procedure to set the threshold for an isolated decision. However, in many real-world decision problems—a bird foraging for food, a market trader deciding whether to keep or sell stocks, a professor going through a pile of job applications or, indeed, a psychology undergraduate doing an experiment for course credits—decisions are not made in isolation; rather, individuals have to make a sequence of decisions. How should one set the threshold in this situation? Is the optimal threshold still given by SPRT? If decision makers accrue a reward from each decision, an ecologically sensible goal for the decision maker may be to maximize the expected reward from these decisions, rather than to minimize the number of samples required to make a decision with a given accuracy (as SPRT does). And for sequences that involve a large number of decisions or sequence of decisions that do not have a clearly defined end point, it would make sense for the decision maker to maximize the *reward rate* (i.e., the expected amount of rewards per unit time). In fact, under certain assumptions, including the assumption that every decision in a sequence has the same difficulty, it can be shown that the two optimization criteria—SPRT and reward rate—result in the same threshold (Bogacz et al., 2006). That is, the decision maker can maximize reward rate by using the SPRT and maintaining an appropriately chosen threshold that remains constant within and across trials. Experimental data suggest that people do indeed adapt their speed and accuracy to improve their reward rate (Bogacz, Hu, Holmes, & Cohen, 2010; Simen et al., 2009). This adaptability seems to be larger for younger than older adults (Starns & Ratcliff, 2010, 2012) and seems to become stronger with practice (Balci et al., 2011) and guidance (Evans & Brown, 2016).

However, maintaining a fixed and time-invariant threshold across a sequence of trials cannot be the optimal solution in many ecologically realistic situations where the difficulty of decisions fluctuates from trial-to-trial. Consider, for example, a situation in which there is very little information or evidence in favor of the different decision alternatives. Accumulating little evidence to a fixed threshold might take a very long time. The decision maker risks being stuck in such an impoverished trial because they are unable to choose between two equally uninformative options, like Buridan’s donkey (see Lamport, 2012), who risks being starved because it is unable to choose between two equally palatable options. Cartwright and Festinger (1943) foresaw this problem and noted that “there is good reason to suppose that the longer the individual stays in the decision region, the weaker are the restraining forces against leaving it” (p. 600). So they speculated that the mean restraining force should be expressed as a decreasing function of time but they were not prepared to make specific assumptions as to the exact nature of this function.

In cases where the restraining force may change with time, the concept of a fixed threshold may be replaced by a time-dependent decision boundary between making more observations and choosing an alternative. A number of recent studies have mathematically computed the shape of decision boundaries that maximize reward rate when decisions in a sequence vary in difficulty and shown that the decision maker can maximize the reward rate by decreasing this decision boundary with time (Drugowitsch, Moreno-Bote, Churchland, Shadlen, & Pouget, 2012; Huang & Rao, 2013; Moran, 2015). It can also be shown that the shape of the boundary that maximizes reward rate depends on the mixture of decision difficulties. Indeed, on the basis of the difficulties of decisions in a sequence, optimal boundaries may decrease, remain constant or increase (Malhotra, Leslie, Ludwig, & Bogacz, 2017; also see subsequent text).^1^

The goal of this study was to test whether, and under what circumstances, humans vary their decision boundaries with time during a decision. More generally, we assessed the relationship between the bounds used by people and the optimal bounds—that is, the boundary that maximizes reward rate. Importantly, we adopt an experimental approach that is firmly rooted in a mathematical optimality analysis (Malhotra et al., 2017) and that allows us to infer the decision boundary relatively directly based on the sequences of evidence samples actually experienced by decision makers.

Previous evidence on whether people change decision boundaries at all during a trial, much less adapt it to be optimal, is inconclusive. Some evidence of time-dependent boundaries was found early on in studies that compared participant behavior with Wald’s optimal procedure. These studies used an expanded-judgment paradigm in which the participant makes their decision based on a sequence of discrete samples or observations presented at discrete times—for example, deciding between two deck of cards with different means based on cards sampled sequentially from the two decks (see, e.g., Becker, 1958; Busemeyer, 1985; Irwin et al., 1956; Manz, 1970; Pleskac & Busemeyer, 2010; Smith & Vickers, 1989; Vickers, 1995). The advantage of this paradigm is that the experimenter can record not only the response time and accuracy of the participant, but also the exact sequence of samples on which they base their decisions. In an expanded-judgment paradigm Pitz, Reinhold, and Geller (1969) found that participants made decisions at lower posterior odds when the number of samples increased. Similar results were reported by Sanders and Linden (1967) and Wallsten (1968). Curiously, participants seemed to be disregarding the optimal strategy in these studies, which was to keep decision boundaries constant. We discuss in the following text why this behavior may be ecologically rational when the participant has uncertainty about task parameters.

The shape of decision boundaries has been also analyzed in a number of experiments using a paradigm where the samples drawn by the participant are implicit, that is, hidden from the experimenter. In these paradigms, the data recorded is limited to the response time and accuracy, so one can distinguish between constant or variable decision boundaries only indirectly, by fitting the two models to the data and comparing them. These tasks generally involve detecting a signal in the presence of noise. Therefore, to distinguish these experiments from the expanded-judgment tasks, we call them *signal detection tasks*. Examples of this paradigm include lexical decisions (Wagenmakers, Ratcliff, Gomez, & McKoon, 2008), basic perceptual discrimination (e.g., brightness; Ratcliff & Rouder, 1998; Ludwig, Gilchrist, McSorley, & Baddeley, 2005), and numerosity judgments (Starns & Ratcliff, 2012). Pike (1968) analyzed data from a number of psychophysical discrimination studies and found that this data is best explained by the accumulator model (Audley & Pike, 1965) if subjects either vary decision bounds between trials or decrease bounds during a trial. Additional support for decreasing boundaries was found by Drugowitsch et al. (2012), who analyzed data collected by Palmer, Huk, and Shadlen (2005). Finally, data from nonhuman primates performing a random dot motion discrimination task (Roitman & Shadlen, 2002) were best fit by a diffusion model with decreasing boundaries (Ditterich, 2006).

In contrast to these studies that found evidence favoring decreasing boundaries, Hawkins, Forstmann, Wagenmakers, Ratcliff, and Brown (2015) analyzed data from experiments on human and nonhuman primates spanning a range of experiments using signal detection paradigms and found equivocal support for constant and decreasing boundaries. They found that overall evidence, especially in humans, favored constant boundaries and that, crucially, experimental procedures such as the extent of task practice seemed to play a role in which option was favored. Therefore, what seems to be missing is a more systematic analysis of the conditions under which people decrease the decision boundary within a trial and understanding why they would do so.

In this study, we took a different approach: Rather than inferring the decision boundaries indirectly by fitting different boundaries to explain RTs and error rates, we used the expanded-judgment paradigm, where the experimenter can observe the exact sequence of samples used by the participant and record the exact evidence and time used to make a decision. This evidence and time should lie on the boundary. This allowed us to make a more direct estimate of the decision boundary used by the participants and compare this boundary with the optimal boundary. We found that, in general, participants modulated their decision boundaries during a trial in a manner predicted by the maximization of reward rate. This effect was robust across paradigms and for decisions that play out over time scales that range from several hundreds of ms to several s. However, there were also systematic deviations from optimal behavior. Much like the expanded-judgment tasks discussed in preceding text, in a number of our experiments participants seemed to decrease their decision boundary even when it was optimal to keep them constant. We mapped these strategies on to the *reward landscape*, predicted by the theoretical model—that is, the variation in reward rate with different settings of the decision boundary. These analyses suggest that participants’ choice of decision boundary may be guided not only by maximizing the reward rate, but also by robustness considerations. That is, they appear to allow for some “error” in their boundary setting due to uncertainty in task parameters and deviate from optimality in a manner that reduces the impact of such error.

Although it has been argued that the results from an expanded-judgment task can be generalized to signal detection paradigms, where sampling is implicit (Edwards, 1965; Irwin & Smith, 1956; Sanders & Linden, 1967; Pleskac & Busemeyer, 2010; Vickers, Burt, Smith, & Brown, 1985), these tasks usually use a slow presentation rate and elicit longer response latencies than those expected for perceptual decisions. It is possible that attention and memory play a different role in decision making at this speed than at faster speeds at which perceptual decisions occur. To address this possibility, we adapted the expanded-judgment task to allow fast presentation rates and consequently elicit rapid decisions.

The rest of the article is split into five sections. First, we summarize the theoretical basis for the relationship between a boundary and reward rate. In the next three sections, we describe a series of expanded-judgment tasks, each of which compares the boundaries used by participants with the theoretically optimal boundaries. In the final section, we consider the implications of our findings as well as the potential mechanisms by which time-varying boundaries may be instantiated. Data from all experiments reported in this article is available online (https://osf.io/f3vhr/).

## Optimal Shape of Decision Boundaries

We now outline how an expanded-judgment task can be mathematically modeled and how this model can be used to establish the relationship between the task’s parameters and decision boundaries that maximize reward rate. We summarize the key results from a theoretical study of Malhotra et al. (2017), provide intuition for them, and state predictions that we tested experimentally.

Consider an expanded-judgment task that consists of making a sequence of decisions, each of which yields a unit reward if the decision is correct. Each decision (or trial) consists of estimating the true state of the world on the basis of a sequence of noisy observations. We consider the simplest possible case in which the world can be in one of two different states, call these *up* or *down*, and each observation of the world can be one of two different outcomes. Each outcome provides a fixed amount of evidence, δ*X*, to the decision maker about the true state of the world:

$$\delta X=\{\begin{array}{ccc} +1 & \text{with probability} & u\\ -1 & \text{with probability} & 1-u \end{array}$$ 1

where *u* is the up-probability that governs how quickly evidence is accumulated and depends on the true state of the world. We assume throughout that *u* ≥ 0.5 when the true state is up and *u* ≤ 0.5 when the true state is down. Note that the parameter *u* will determine the difficulty of a decision—when *u* is close to 0.5 the decision will be hard while when *u* is close to 0 or 1 the decision will be easy.

**Figure 1 fig1:**
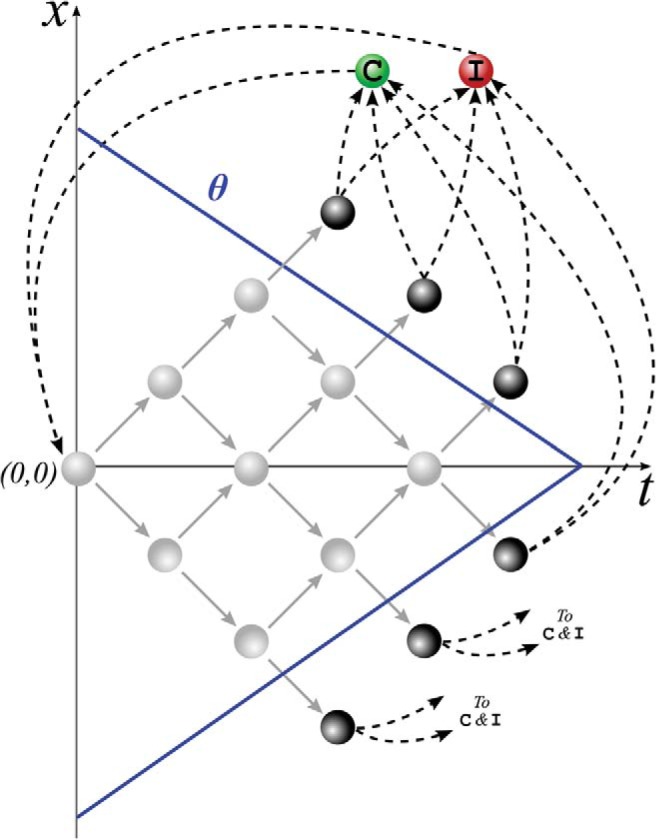
Evidence accumulation and decision making as a Markov decision process: States are shown by circles, transitions are shown by arrows, and actions are shown by color of the circles. The solid (blue) line labeled θ indicates a hypothetical decision boundary. The policy that corresponds to the boundary is indicated by the color of the states. Black circles indicate the action go, whereas gray circles indicate wait. Dashed lines with arrows indicate transitions on go, whereas solid lines with arrows indicate transitions on wait. The rewarded and unrewarded states are shown as **C** and **I**, respectively (for *correct* and *incorrect*). See the online article for the color version of this figure.

Figure 1 illustrates the process of making a decision in this expanded-judgment task. As assumed in sequential sampling models, decision making involves the accumulation of the probabilistic evidence, so let *x* be the cumulative evidence, that is, the sum of all δ*X* outcomes. The accumulation continues till *x* crosses one of two boundaries θ corresponding to the options, so that the decision maker responds up when *x* > θ, and down when *x* < −θ. During the expanded-judgment task described above, the state of the decision maker, at any point of time, is defined by the pair (*t*, *x*), where *t* is the number of observations made. In any given state, the decision maker can take one of two actions: (a) make another observation—we call this action *wait*, or (b) signal their estimate of the true state of the world—we call this the action *go*. As shown in Figure 1, taking an action wait can lead to one of two transitions: the next observed outcome is +1; in this case, make a transition to state (*t* + 1, *x* + 1), or the next observed outcome is −1; in this case, make a transition to state (*t* + 1, *x* − 1). Similarly, taking the action go can also lead to one of two transitions: (a) The estimated state is the true state of the world; in this case, collect a reward and make a transition to the correct state, or (b) the estimated state is not the true state of the world; in this case, make a transition to the incorrect state. After making a transition to a correct or incorrect state, the decision maker starts a new decision, that is, returns to the state (*t*, *x*) = (0, 0) after an intertrial delay *D*_*C*_ following a correct choice and *D*_*I*_ following the incorrect choice.[Fig-anchor fig1]

These set of state-action pairs and transitions between these states defines a Markov decision process (MDP) shown schematically in Figure 1. In this framework, any decision boundary that is a function of time, θ = *f*(*t*), can be mapped to a set of actions, such that action wait is selected for any state within the boundaries, and action go is selected for any state on or beyond the boundaries. The mapping that assigns actions to all possible states is called a *policy* for the MDP.

We assume that a decision maker wishes to maximize the reward rate (i.e., the expected number of rewards per unit time). The reward rate depends on the decision boundary: If the boundary is too low, the decision maker will make errors and miss possible rewards, but if it is too high, each decision will take a long period, and the number of reward per unit of time will also be low.

The policy that maximizes average reward can be obtained by using a dynamic programming procedure known as *policy iteration* (Howard, 1960; Puterman, 2005; Ross, 1983). Several recent studies have shown how dynamic programming can be applied to decision-making tasks to get a policy that maximizes reward rate (see, e.g., Drugowitsch et al., 2012; Huang & Rao, 2013; Malhotra et al., 2017). We now summarize how the optimal shape of decision boundary depends on task’s parameters on the basis of the analysis given in Malhotra et al. (2017). Let us first consider a class of tasks in which the difficulty of the decisions is fixed. That is, evidence can point either toward up or down, but the quality of the evidence remains fixed across decisions: $u\;\in \;\{\frac{1}{2}-\epsilon ,\;\frac{1}{2}+\epsilon \}$, with ϵ corresponding to the drift. The drift can take values in range $\epsilon \;\in [0,\;\frac{1}{2}]$ and it determines the difficulty of each trial with higher drift corresponding to easier trials. For single-difficulty tasks, ϵ remains fixed across trials.

In the single-difficulty tasks, reward rate can be optimized by choosing a policy such that the decision boundary remains constant during each decision. Intuitively, this is because the decision maker’s estimate of the probability that the world is in a particular state depends only on integrated evidence *x*, but not on time elapsed within the trial *t*. Therefore, the optimal action to take in each state only depends on *x* but not *t*, so go actions are only taken if *x* exceeds a particular value, leading to constant boundaries.

**Figure 2 fig2:**
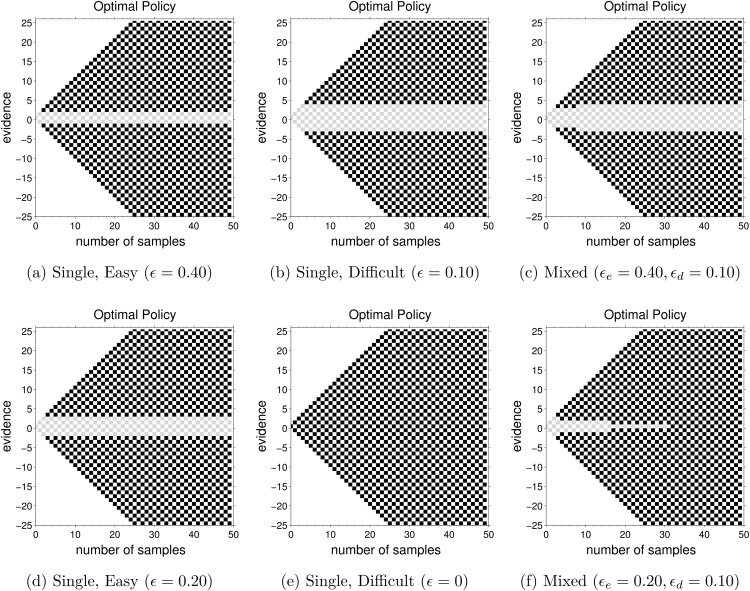
Optimal policies for single and mixed-difficulty tasks. Black squares indicate states of the MDP where the optimal action is to go—that is, choose an alternative—whereas gray squares indicate states where the optimal action is to wait—that is, collect more evidence. In each row, the two panels on the left show optimal policies for single-difficulty tasks with two different levels of difficulty and the right-most panel shows optimal policy for mixed-difficulty task obtained by mixing the difficulties is the two left-hand panels. The intertrial intervals (ITIs) were *D*_*C*_ = *D*_*I*_ = 70 for the top row (Panels a through c) and *D*_*C*_ = *D*_*I*_ = 50 for the bottom row (Panels d through f).

The optimal height of the decision boundary in the single-difficulty tasks depends on task difficulty in a nonmonotonic way. For very easy tasks (ϵ close to $\frac{1}{2}$), each outcome is a very reliable predictor of the state of the world, so very few outcomes need to be seen to obtain an accurate estimate of the state of the world (Figure 2a). As the difficulty increases, more outcomes are required, and the optimal boundary increases (compare Figures 2a and 2b). However, when the task becomes very difficult (ϵ close 0), there is little benefit in observing the stimulus at all, and for ϵ = 0 the optimal strategy is not to integrate evidence at all, but guess immediately, that is, θ = 0 (compare Figures 2d and 2e).[Fig-anchor fig2]

Let us now consider a mixed-difficulty task, in which half of the trials are easy with drift ϵ_*e*_ and the other half of the trials are difficult with drift ϵ_*d*_, where ϵ_*e*_ > ϵ_*d*_. We assume that during mixed-difficulty tasks, the decision maker knows that there are two levels of difficulty (either through experience or instruction), but does not know if a particular trial is easy or difficult. Indeed, a key assumption of the underlying theory is that the difficulty level is something the decision maker has to infer during the accumulation of evidence.

In mixed-difficulty tasks, reward rate is optimized by using boundaries that may decrease, increase or remain constant based on the mixture of difficulties. Intuitively, this is because the decision maker’s estimate of the probability that the world is in a particular state, given the existing evidence, depends on their inference about the difficulty of the trial. Time becomes informative in mixed-difficulty tasks because it helps the decision maker infer whether a given trial is easy or difficult and hence the estimate of the true state of the world depends not only on the evidence, *x*, but also on the time, *t*. The optimal decision maker should begin each decision trial assuming the decision could be easy or difficult. Therefore, θ at the beginning of the trial should be in between the optimal boundaries for the two difficulties. As they make observations, they will update their estimate of the task difficulty. In particular, as time within a trial progresses, and the decision boundary has not been reached, the estimated probability of the trial being difficult increases and the decision boundary moves toward the optimal boundary for the difficult trials.

The above principle is illustrated in Figures 2c and 2f showing optimal boundaries for two sample mixed-difficulty tasks. Figure 2f shows the optimal boundary for a task in which half of the trials have moderate difficulty and half are very difficult (the optimal bounds for single-difficultly tasks with corresponding values of drift are shown in Figures 2d and 2e). As the time progresses the optimal decision maker infers that a trial is likely to be very difficult, so an optimal strategy involves moving on to the next trial (which may be easier), that is, decreasing the decision boundary with time in the trial.

In contrast, when the boundary for the difficult task is higher than the easy task (the difficult task is not extremely hard; Figures 2a and 2b), the optimal boundary in the mixed-difficulty task will again start at a value in-between the boundaries for the easy and difficult tasks and approach the boundary for the difficult task (Figure 2c). In this case, the boundary for the difficult task will be higher than the easy task meaning that the optimal boundary will increase with time.

In summary, the mathematical model makes three key predictions about the normative behavior: (a) optimal decision boundaries should stay constant if all decisions in a sequence are of the same difficulty, (b) it is optimal to decrease decision boundaries if decisions are of mixed difficulty and some decisions are extremely difficult (or impossible), and (c) it may be optimal to keep decision boundaries constant or even increase them in mixed-difficulty tasks where the difficult decision is not too difficult. In the next three sections, we compare human behavior with these normative results.

## Experiment 1

To compare human behavior with the normative behavior described previously, we designed an experiment that involved an evidence-foraging game which parallels the expanded-judgment task described in the previous section. We modeled this evidence-foraging game on previous expanded-judgment tasks, such as Irwin et al. (1956) and Vickers, Burt, et al. (1985), where participants are shown a sequence of discrete observations and required to judge the distribution from which these observations were drawn. We modified these expanded-judgment paradigms so that (a) the observations could have only one of two values (i.e., drawn from the Bernoulli distribution), (b) the reward-structure of the task was based on performance, and (c) the task had an intrinsic speed–accuracy trade-off. We introduced a speed–accuracy trade-off by using a fixed-time blocks paradigm: the experiment was divided into a number of games, the total duration for each game was fixed, and participants could attempt as many decisions as they like during this period. Therefore, if a participant takes a very long time for each decision they are likely to be accurate, but will not be able to complete many decisions during a game. If a participant decides very quickly, they are likely to perform worse in terms of accuracy, but will have more reward “opportunities” during the game. The goal of the participants was to collect as much reward as possible during each game, so they need to find a balance between these two strategies.

In this expanded judgment task, we are able to record the exact sequence of stimuli presented to the participants and the position in state-space (*t*, *x*) at which participants made their decisions. Based on the location of these decisions, we inferred how the decision boundary for a participant depended on time. According to the above theory, the optimal decision boundary should be independent of time in single-difficultly tasks, but could vary with time during mixed-difficulty tasks. By comparing the inferred decision boundary with optimal boundaries in each type of task, we assessed whether participants adjusted their decision boundaries to maximize reward rate.

### Method

#### Description of task

Twenty-four participants from the university community were asked to play a set of games on a computer. The number of participants was chosen to give a sample size that is comparable to previous human decision-making studies and kept constant during all of our experiments.^2^ Each game lasted a fixed duration and participants made a series of decisions during this time. Correct decisions led to a reward and participants were asked to maximize the cumulative reward. The game was programmed using Matlab® and Psychtoolbox (Brainard, 1997; Kleiner et al., 2007; Pelli, 1997) and was played using a computer keyboard. The study lasted approximately 50 min, including the instruction phase and training.

**Figure 3 fig3:**
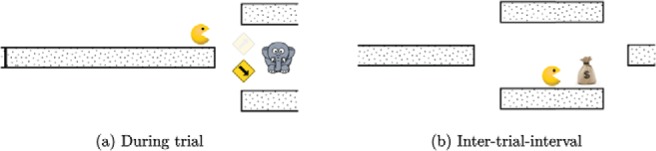
Two screenshots of the display during the experiment. The left panel shows the display during the evidence-accumulation phase of a trial. Participants chose whether Pacman goes up or down after seeing a sequence of cues (arrows) pointing up or down. The elephant next to the arrow indicates that this is an easy game, so the arrow points to the reward-holding path with probability 0.70. The right panel shows a screenshot during the intertrial interval (ITI). The participant has chosen the lower path and can now see that this was the correct (rewarded) decision. See the online article for the color version of this figure.

During each game, participants were shown an animated creature (Pacman) moving along a path (see Figure 3). A trial started with Pacman stationary at a fork in the path. At this point Pacman could jump either up or down and the participant made this choice using the *up* or *down* arrow keys on the keyboard. One of these paths contained a reward, but the participant could not see this before making the decision. Participants were shown a sequence of cues and they could wait and watch as many cues as they wanted before making their choice. The display also showed the total reward they accumulated in the experiment and a progress bar showing how much time was left in the current game.[Fig-anchor fig3]

Once the participant indicated their choice, an animation showed Pacman moving along the chosen path. If this path was the rewarded one, a bag with a $ sign appeared along the path (right panel in Figure 3). When Pacman reached this bag, the reward was added to the total and Pacman navigated to the next fork and this started the next trial. If the participant chose the unrewarded path, the money bag appeared along the other path.

The intertrial interval (ITI) started as soon as the participant indicated their choice. We manipulated the ITI for correct and incorrect decisions by varying Pacman’s speed. Participants were told that Pacman received a “speed-boost” when it ate the money bag so that ITI for correct decisions was smaller than that for incorrect decisions. Values for all parameters used during the game are shown in Table 1.[Table-anchor tbl1]

#### Cue stimuli

When Pacman reached a fork, cues were displayed at a fixed rate, with a new cue every 200 ms. We call this delay the interstimulus interval (ISI). During these 200 ms, the cue was displayed for 66 ms, followed by 134 ms of no cue. Each cue was the outcome of a Bernoulli trial and consisted of either an upward or a downward pointing arrow. This arrow indicated the rewarded path with a particular probability.

Next to the cues, participants were shown a picture of either an elephant or a penguin. This animal indicated the type of game they were playing. One of the two animals provided cues with a probability 0.70 of being correct, while the other animal provided cues with a probability 0.50 of being correct. Thus, the two animals mapped to the two single-difficultly conditions—easy (with ϵ = 0.20) or difficult (with ϵ = 0)—shown in Figure 2d and 2e. The mapping between difficulties and animals was counterbalanced across participants.

We chose the values of up-probability so that the optimal decision boundaries in the mixed-difficulty case have the steepest slope, making it easier to detect if participants decrease decision boundaries. The theory in the previous section shows that decision boundaries decrease only when the difficult decisions are extremely difficult. In the experiment we set the up-probability for difficult condition to the extreme value of 0.5, that is, ϵ_*d*_ = 0; therefore, the cues do not give any information on the true state of the world. Using this value has two advantages: (a) it leads to optimal decision boundaries in mixed-difficulty games with the steepest decrease in slope, and (b) it makes it easier for participants to realize that the optimal boundary in the difficult condition is very low (in fact, the optimal strategy for difficult games is to guess immediately). Optimal boundaries should also decrease (although with a smaller slope) when decisions are marginally easier (e.g., ϵ_*d*_ = 0.03). But we found that participants frequently overweight evidence given by these low probability cues (perhaps analogous to the overweighting of small probabilities in other risky choice situations, e.g., Tversky & Kahneman, 1992; Gonzalez & Wu, 1999) and need a large amount of training to establish the optimal behavior in such extremely difficult (but not impossible) games. In contrast, when ϵ_*d*_ = 0, participants could learn the optimal strategy difficult games with a small amount of training.

The experiment consisted of three types of games: *easy games*, where only the animal giving 70% correct cues appeared at each fork; *difficult games*, where only the animal giving the 50% cues appeared at the fork; and *mixed games*, where the animal could change from one fork to the next. Participants were given these probabilities at the start of each game and also received training on each type of game (see Structure of Experiment section). Importantly, during mixed games participants were shown a picture of a wall instead of either animal and told that the animal was hidden behind this wall. That is, other than the cues themselves, they received no information indicating whether a particular trial during a mixed game was easy or difficult so that they had to infer the type of trial solely on the basis of these cues. This corresponds to the mixed-difficulty task shown in Figure 2f.

#### Reward structure

Participant reimbursement was broken down into three components. The first component was fixed and every participant received (approx $7.5) for taking part in the study. The second component was the money bags accumulated during the experiment. Each money bag was worth (approx 2 cents) and participants were told that they could accumulate up to (approx $6) during the experiment. The third component was a bonus prize of (approx $25) available to the participant who accumulated the highest reward during the study. Participants were not told how much other participants had won until after they took part in the study.

#### Structure of experiment

The experiment was divided into a training phase and a testing phase. Participants were given training on each type of game. The duration of each training game was 150 s. This phase allowed participants to familiarize themselves with the games and probability of cues as well as understand the speed–accuracy trade-off for each type of games. The reward accumulated during the training phase did not count toward their reimbursement.

The testing phase consisted of six games, two of each type. Participants were again reminded of the probabilities of cues at the start of each game. The order of these games was counterbalanced across participants so that each type of game was equally likely to occur in each position in the sequence of games. The duration of the easy games was 240 s, whereas the difficult and mixed games lasted 300 s each. The reason for different durations for different types of games was that we wanted to collect around the same amount of data for each condition. Pilot studies showed that participants generally have faster reaction time (RT) during the easy games (see following Results section). Therefore, we increased the length of the difficult and mixed blocks. By using these durations, participants made approximately 70 to 90 choices during both easy and mixed conditions. In the middle of each game, participants received a 35 s break.

#### Eliminating nondecision time

We preprocessed the recorded data to eliminate nondecision time—the delay between making a decision and executing a response. As a result of this nondecision time, the data contained irrelevant stimuli that were presented after the participant had made their decision. To eliminate these irrelevant stimuli, we estimated the nondecision time for each participant on the basis of their responses during the easy games. Appendix A illustrates the method in detail; the key points are summarized briefly in the following text.

For each participant, we reversed the sequence of stimuli and aligned them on the response time. Let us call these ordered sequence (*s*_1_^*i*^, *s*_2_^*i*^, . . . , *s_T_^i^*), where *i* is the trial number and (1, 2, . . . , *T*) are the stimulus indices before the response. Each stimulus can be either up or down, that is, *s_t_^i^* ∈ {up, down}. At each time step, we estimated the correlation (across trials) between the observing a stimulus in a particular direction and making a decision to go in that direction. That is, we computed *p*_*t*_ at each stimulus index, *t* ∈ {1, 2, . . . , *T*}, as the fraction of trials where the response *r^i^* ∈ {up, down} is the same as *s_t_^i^*. So, for each participant, the values (*p*_1_, *p*_2_, . . . , *p*_*T*_) serve as an estimate of the correlation between the stimulus at that index and the response.

**Figure A1 fig12:**
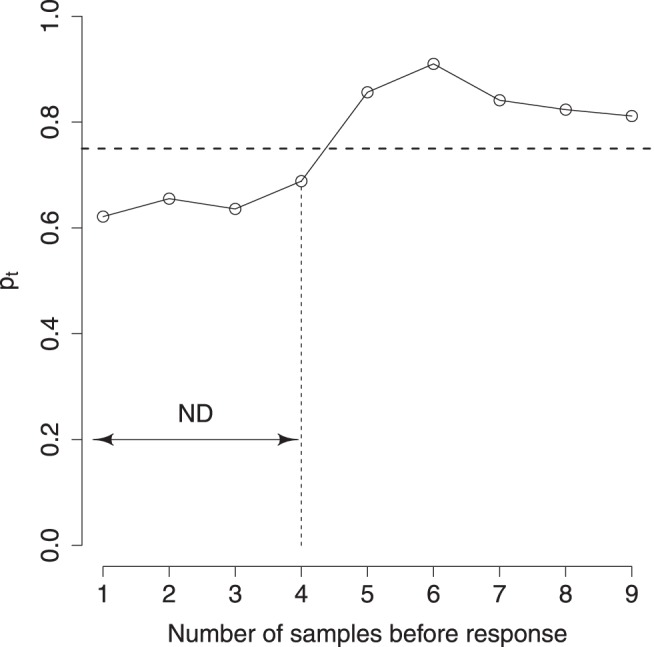
The plot shows the proportion of trials at each time step before the response (for a particular participant and condition) that are in the same direction as the response. The dashed horizontal line represents a threshold on this proportion used to compute the nondecision time. The dashed vertical line shows the last time-step where this proportion was below the threshold.

If stimuli at a particular index, *t*, occurred after the decision, that is, during the nondecision time, we expected them to have a low correlation with response and consequently *p*_*t*_ to be below the drift rate, 0.70. We determined the first index in the sequence with *p*_*t*_ larger than 0.75; that is, the first index with more than 75% of stimuli in the same direction as the response. This gave us an estimate of the number of stimuli, *ND*, that fall in the nondecision period. We used this estimate to eliminate the stimuli, *s*_1_, . . . , *s*_*ND*_ from each recorded sequence for the participant. See Figure A1 in Appendix A.

For 21 out of 24 participants, we estimated *ND* = 1, that is, a nondecision delay of approximately 200 ms. For two subjects the nondecision delay was two stimuli and for one participant no stimuli were excluded.

#### Exclusion of participants

To ensure that each participant understood the task, we conducted a binomial test on responses in the easy and mixed-difficulty games. This test checked whether the number of correct responses during a game were significantly different from chance. Two participants failed this test during mixed-difficulty games and were excluded from further analysis.

#### Analysis method

We now describe how we estimated the decision boundary underlying each participant’s decisions. In signal-detection paradigms, the experimenter cannot observe the exact sequence of samples based on which the participant made their decision. Therefore, parameters like boundary are obtained by fitting a sequential sampling or accumulator model to the RT and error distributions. In contrast, the expanded-judgment paradigm allows us to observe the entire sequence of samples used to make each decision. Therefore, our analysis method takes into account not only the evidence and time at which the decision (‘up’/‘down’) was made, but also the exact sequence of actions (*wait–go*) in response to the sequence of cues seen by the participant. It also takes into account the trial-to-trial variability in the behavior of participants: even when participants saw the exact same sequence of cues, they could vary their actions from one trial to next.

If a participant makes a decision as soon as evidence crosses the boundary, the value of time and evidence, (*t*, *x*) during each decision should lie along this boundary. Therefore, one way to recover this boundary is by simply fitting a curve through the values of (*t*, *x*) for all decisions in a block. However, note that participants show a trial-to-trial variability in their decision making. Sequential sampling models account for this trial-to-trial variability by assuming noisy integration of sensory signals as well as variability in either drift, starting point or in threshold (see Ratcliff, 1978; Ratcliff & Smith, 2004). We chose to model this variability by assuming there is stochasticity in each *wait–go* decision. That is, instead of waiting when evidence was below the boundary and going as soon as evidence crossed the boundary, we assumed that a participant’s decision depended on the outcome of a random variable, with the probability of the outcome depending on the accumulated evidence and time.

Specifically, we define two predictor variables—the evidence accumulated, *X* = *x*, and the time spent in the trial, *T* = *t* – and a binary response variable, *A* ∈ *wait, go*. The probability of an action can be related to the predictor variables using the following logistic regression model:

$$\log\frac{P(A=go)}{\mathbb{P}(A=wait)}=\beta_{0}+\beta_{T}*T+\beta_{X}*X$$ 2

where β_*T*_ and β_*X*_ are the regression coefficients for time and evidence, respectively, and β_0_ is the intercept. Given the triplet (*X*, *T*, *A*) for each stimulus in each trial, we estimated for each type of game and each participant the β^0,β^T and β^X that maximized the likelihood of the observed triplets.

**Figure 4 fig4:**
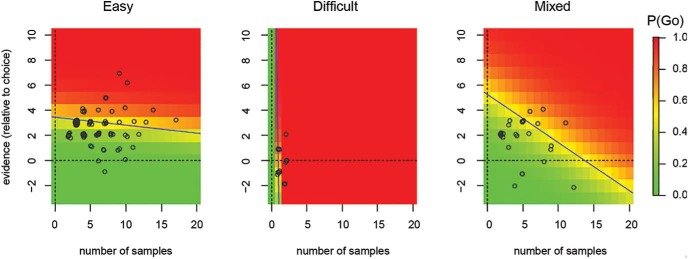
The decisions made by a subject during Experiment 1 and the inferred boundaries based on these decisions. Each scatter-plot shows the values of evidence and time where the subject made decisions during a particular game (only easy trials considered during mixed game). These values have been slightly jittered for visualization. The heat-map shows the $P(Go|X_{t}=x)$ for each *x* and *t* inferred using logistic regression. The solid line shows a “line of indifference” where $P(Go|X_{t}=x)=P(Wait|X_{t}=x)$ and serves as a proxy for the subject’s boundary (see Appendix B). See the online article for the color version of this figure.

Figure 4 shows the results of applying the above analysis to one participant. The data are split according to condition – easy, difficult or mixed. Each circle shows the end of a random walk (sequence of stimuli) in the time-evidence plane. These random walks were used to determine the (maximum likelihood) regression coefficients, β^0,β^X and β^T, as outlined above. These estimated coefficients are then used (Equation 2) to determine the probability of *go*ing at each *x* and *t*, which is shown as the heat-map in Figure 4.[Fig-anchor fig4]

This heat-map shows that, under the easy condition, this participant’s probability of *go*ing strongly depended on the evidence and weakly on the number of samples. In contrast, under the difficult condition, the participant’s probability of *go*ing depends almost exclusively on the number of samples—most of their decisions are made within a couple of samples and irrespective of the evidence. Under the mixed condition, the probability of *go*ing is a function of both evidence and number of samples.

Since we were interested in comparing the slopes of boundaries during easy and mixed conditions, we determined a *line of indifference* under each condition, where P(A=go)=P(A=wait), that is, the participant was equally likely to choose actions *wait* and *go*. Substituting in Equation 2 gives the line:

X=−β^Tβ^X∗T−β^0β^X 3

with slope −β^Tβ^X and intercept as −β^0β^X. We used the slope of this line as an estimate for the slope of the boundary. Appendix B reports a set of simulations that tested the validity of this assumption and found that there is a systematic relationship between this inferred slope and the true slope generating decisions. Importantly, these simulations also demonstrate that even if the variability in data is due to noisy integration of sensory signals (rather than trial-to-trial variability in decision boundary), this inferential method still allows us to make valid comparisons of slopes of boundaries in easy and mixed games.

Each panel in Figure 4 also shows the line of indifference for the condition. The slope of the line of indifference is steepest under the difficult condition followed by the mixed condition and most flat for the easy condition. Note that for the mixed condition, we only considered the “easy” trials—that is, trials showing cues with correct probability = 0.70. This ensured that we made a like-for-like comparison between easy and mixed conditions.

A quantitative comparison of slopes between conditions can be made by taking the difference between slopes. However, a linear difference is inappropriate as large increasing slopes are qualitatively quite similar to large decreasing slopes—both indicate a temporal, rather than evidence-based boundary (e.g., the difficult condition in Figure 4). Therefore, we compared slopes in the mixed and easy conditions by converting these slopes from gradients to degrees and finding the circular difference between slopes:

$$\Delta m=((m_{e}-m_{m}+90)\;mod\;180)-90$$ 4

where *m*_*e*_ and *m*_*m*_ are the slopes in easy and mixed conditions, respectively; Δ*m* is the difference in slopes and *mod* is the modulo operation. Equation 4 ensures that the difference between slopes is confined to the interval [−90, +90] degrees and large increasing slopes have a small difference to large decreasing slopes.

The above analysis assumes that evidence accumulated by a participant mirrors the evidence presented by the experimenter—so there is no loss of evidence during accumulation and the internal rate of evidence accumulation remains the same from one trial to next. In Appendix C we performed simulations to verify that inferences using the above analysis remain valid even when there is loss in information accumulated and when the drift rate varied from one trial to next.

### Results

**Figure 5 fig5:**
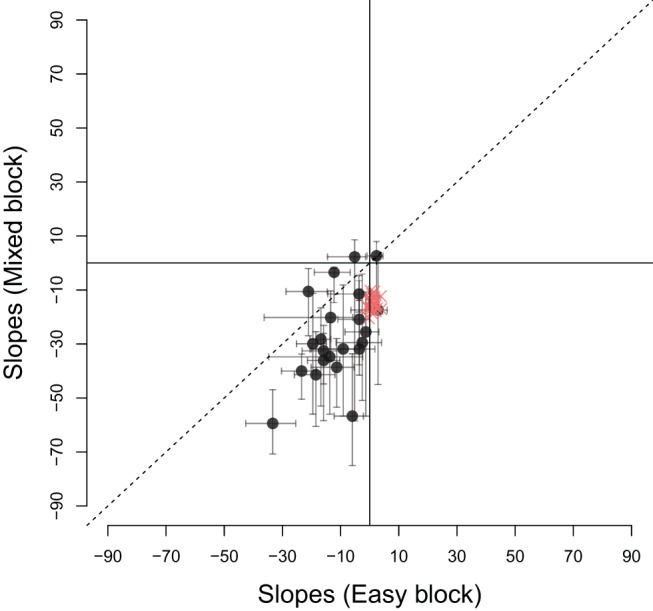
Each circle (black) compares the estimated slope in easy and mixed games for 1 participant. Circles below the dashed line were participants who had a larger gradient of the inferred boundary during the mixed games as compared to the easy games. Error bars indicate the 0.95 percentile bootstrapped confidence intervals for the estimated slopes. Crosses (red) show 24 simulated participants—decisions were simulated using a rise-to-threshold model with optimal boundaries shown in Figures 2d and 2f. See the online article for the color version of this figure.

The mean RTs during easy, difficult and mixed games were 1444 ms (*SEM* = 23 ms), 1024 ms (*SEM* = 47ms) and 1412 ms (*SEM* = 22ms), respectively, where *SEM* is the within-subject standard error of the means. Note that ‘RT’ here refers to ‘decision time,’ that is, the raw response time minus the estimated nondecision time. As noted above, the nondecision time for most participants was approximately 200 ms. Figure 5 compares the slopes for the lines of indifference in the easy and mixed games (black circles). Error bars indicate the 0.95 percentile confidence interval.^3^ Like the participant shown in Figure 4 the estimated slope for most participants was more negative during the mixed games than during easy games, falling below the identity line. A paired *t* test on the difference in slopes in the two conditions (using Equation 4) confirmed that there was a significant difference in the slopes (*t*(21) = 5.24, *p* < 0.001, *m* = 15.94, *d* = 1.20), indicating that the type of game modulated how participants set their decision boundary.[Fig-anchor fig5]

Figure 5 also shows the relationship between the slopes of easy and mixed games for 24 simulated participants (red crosses) who optimize the reward rate. Each of these participants had slopes of boundary calculated using dynamic programming (Malhotra et al., 2017) and made decisions based on a noisy integration of evidence to this optimal boundary. The slopes in each condition were then inferred using the same procedure as for our real participants. These optimal participants, like the majority of participants in our study, had a larger (negative) slope in the mixed condition than the easy condition. However, in contrast to the optimal participants, the majority of participants also exhibited a negative slope during the *easy* games, indicating that they lowered their decision boundary with time during this condition. A *t* test confirmed that the slope during easy condition was less than zero (*t*(21) = −5.51, *p* < 0.001, *m* = −11.47). Participants also showed substantial variability in the decision boundary in the easy condition, with slopes varying between 0 and 45 degrees.

An alternative possibility is that participants change their decision boundary during the experiment, adopting a higher (but constant) boundary toward the beginning and lowering it to different (constant) boundary during the experiment. In order to check for this possibility, we split the data from each condition into two halves and checked whether the mean number of samples required to make a decision changed from the first half to second half of the experiment. During easy games, we found that participants observed 7.5 and 6.8 samples, on average, during the first and second half of the experiment, respectively. During mixed games, these mean observations changed to 6.8 and 6.2 samples, on average, during the first and second half of the experiment. A two-sided paired *t* test which examined whether the mean number of samples were different in the two halves of the experiment found no significant difference in either the easy games (*t*(21) = 1.76, *p* = 0.09, *m* = 0.73) or in the mixed games (*t*(21) = 1.40, *p* = 0.18, *m* = 0.64).^4^

We checked the robustness of these results by performing a model comparison exercise, pitting a time-varying decision boundary against a fixed boundary model. The latter simply involves a logistic regression in which the decisions to *wait* or *go* were based on evidence only. The full details of this model comparison procedure and results are described in Appendix D. Based on a comparison of Bayesian Information Criteria (Schwarz, 1978; Wagenmakers, 2007), the time-varying model provided a better account of the behavior of 15 out of 22 participants in mixed-difficulty games. For 3 participants, the evidence was ambiguous and for the remaining 4 participants the simpler, fixed boundary model won. In easy games, the model using time as a predictor was better at accounting for data from 13 participants while the simpler model performed better to data for 8 participants.

**Figure 6 fig6:**
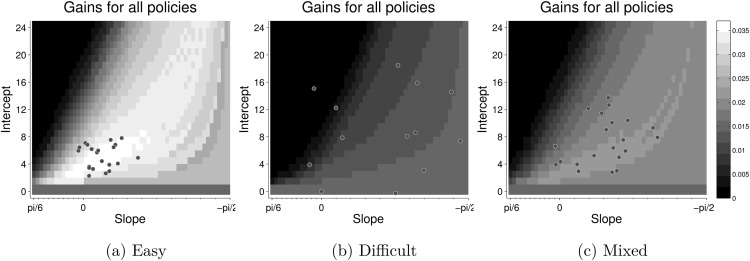
The reward rates for different decision boundaries. In each panel, the slope and intercept determine a linear boundary. The actions of all states below the boundary are set to wait and all states above are set to go. The heat-map in each panel shows the reward rate for each threshold. The circles show the inferred boundaries used by the participants in Experiment 1.

In order to understand why participants decrease the decision boundary in easy games and why different participants show a large variation in their choice of boundary, we computed the reward rate accrued by each participant’s choice of boundary and compared it to the reward rate for the optimal policy. This gave us the cost of setting any nonoptimal decision boundary. Figure 6 shows the landscape (heat-map) of the reward rate for each type of game for a host of different boundaries, defined by different combinations of intercepts and slopes. The circles indicate the intercepts and slopes of the inferred line of indifference of each participant.[Fig-anchor fig6]

Notice, in particular, the landscape for the easy games. Even though the peak of this landscape lies at the policy with zero slope (flat bounds), there is a “ridge” of policies on the landscape where the reward rate is close to optimal. The policies chosen by most participants in Experiment 1 seem to lie along this ridge—even though participants do not necessarily choose the optimal policy, they seem to be choosing policies that are close to optimal. A similar pattern holds in the mixed games. In contrast, during difficult games, the average reward is low, irrespective of the policy. Correspondingly, there is a large variability in the policies chosen by participants. We examine the effect of reward landscape on the policies chosen by participants in more detail at the end of Experiment 2.

## Experiment 2

Experiment 1 established that people modulate their decision boundary based on task difficulty and variations in the reward landscape. However, our experimental paradigm—effectively an expanded-judgment task—is clearly very different from the dominant, typically signal-detection paradigms used to test rise-to-threshold models and time-varying boundaries (e.g., Britten, Shadlen, Newsome, & Movshon, 1992; Palmer et al., 2005; Ludwig, 2009; Starns & Ratcliff, 2012). In our paradigm, response times in mixed games were generally between 1 and 2 s, whereas in the perceptual decision-making literature, RTs are typically between 0.5-1 s (Palmer et al., 2005). It is possible that at this speed participants do not, or cannot, modulate their decision boundaries and instead adopt suboptimal fixed thresholds.

Our aim in Experiment 2 then was to replicate and extend our findings to a more rapid task, where RTs were similar to a signal-detection paradigm. More generally, we tested the robustness and generality of the results from the expanded-judgment task of Experiment 1 by introducing different (i) stimulus materials, (ii) ISIs and (iii) ITIs. The variation in ISI was designed to induce more rapid decision making (with RTs typically < 1*s*). Since the optimal policies are computed on a relative time scale (based on a unit ISI), we can scale both the interstimulus and ITI without affecting the optimal policy, but reducing the RT. The variation in ITI (specifically: for correct decisions, *D*_*C*_) was introduced to manipulate the reward landscape, without affecting the *optimal policy*. Bogacz et al. (2006) have previously shown that the optimal policy is invariant to change in *D*_*C*_ for single-difficultly games. Malhotra et al. (2017) showed that this result generalizes to the mixed-difficulty scenario: optimal policy for mixed-difficulty games depends only on the ITI for incorrect decisions, *D*_*I*_, but is independent of the ITI for correct decisions, *D*_*C*_, as long as *D*_*C*_ < *D*_*I*_. If participants were optimizing the reward rate, they should not change their decision boundary with a change in *D*_*C*_. However, as we will see below, changing *D*_*C*_ does affect the wider reward landscape around the optimal policy and we explored to what extent participants were sensitive to this change.

Permutations of varying these two parameters leads to four experiments, which we have labeled Experiments 2a–2d. The values of parameters for each experiment are shown in Table 2. Experiment 2a was a replication of Experiment 1 with exactly the same parameters, but using the new paradigm (described below). In Experiment 2b, we scaled the ISI and ITI to elicit rapid decisions but kept all other parameters the same as Experiment 2a. In Experiment 2c, we increased the inter-trial-interval for correct responses to match that for incorrect responses. All other parameters were kept same as Experiment 2a. Finally, in Experiment 2d, we scaled ISI and ITI to elicit rapid decisions and also matched ITIs for correct and incorrect decisions.[Table-anchor tbl2]

Like Experiment 1, 24 healthy adults between the age of 18 and 35 from the university community participated in each of these experiments, with no overlapping participants between experiments.

### Method

Decreasing the ISI increases two sources of noise in the experiment: (i) noise due to variation in attention to cues (i.e., there is a greater likelihood of participants “missing” samples when they are coming in faster; ii) noise due to visual interference between consecutive cues in the same location. The second source of noise is particularly challenging for our purposes. That is, the analysis presented here assumes that each evidence sample is processed independently. However, if we were to present a sequence of cues in rapid succession, it is clear that, due to the temporal response properties of the human visual system, successive cues could “blend in” with each other (Georgeson, 1987). As a result, we could not simply speed-up the presentation of the arrow cues in the Pacman task. We adapted the original task from Experiment 1 to another evidence-foraging game that retained the structure of the paradigm and that allowed for systematic variation of the various parameters of interest (i.e., interstimulus and ITIs).

**Figure 7 fig7:**
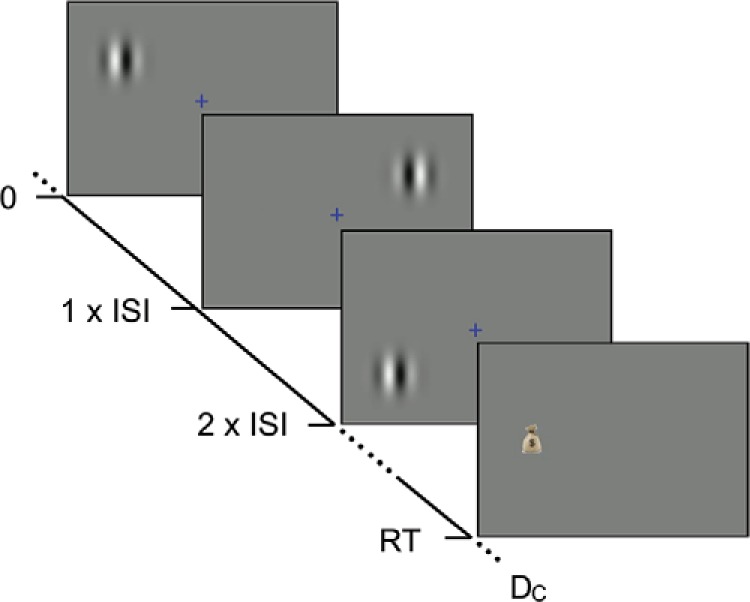
An illustration of the paradigm for Experiments 2a through 2d. During each trial, participants chose left or right based on a sequence of cues. Each cue was a Gabor pattern displayed (for a fifth of interstimulus interval [ISI]) in one of four possible locations, equidistant from the fixation cross. If the decision was correct (as in this example), a money bag was displayed on the chosen side of the fixation cross and the participant waited for the duration *D*_*C*_ before starting the next trial. If the decision was incorrect, no money bag was displayed and the participant waited for the duration *D*_*I*_ before starting the next trial. RT = Reaction time. See the online article for the color version of this figure.

Participants were again asked to maximize their cumulative reward by making correct decisions in a game. But now, during each trial participants focused on a fixation cross in the middle of the screen with gray background^5^ and were told that a reward was either on the left or the right of the fixation cross. In order to make their choice, participants were shown cues that could appear either to the left or right of the fixation cross. In order to minimize interference (see below) cues could appear in two alternative locations on each side – ‘left-up’ or ‘left-down’ on the left and ‘right-up’ or ‘right-down’ on the right. A cue appeared on the same side as the reward with a given probability. Participants were given this probability at the beginning of the game. For single-difficultly games, they were told that this probability was the same $\left(\frac{1}{2}\pm 0.22\right)$ for all trials within this game. For the mixed-difficulty games, they were told that a particular trial during the game could give cues with one of two different probabilities $(\frac{1}{2}\pm 0.22$ or $\frac{1}{2}\pm 0)$ and they were given these possible probabilities at the start of each game (i.e., block). Participants were again told that they could see as many cues as they wanted during a trial before making a decision, but the total duration of the game was fixed. Figure 7 shows an example trial in which the participant makes the decision to go left after observing a series of cues.[Fig-anchor fig7]

Each cue was a Gabor pattern (sinusoidal luminance grating modulated by a 2D Gaussian window). We designed these cue patterns to minimize interference between consecutive patterns. The integration period of early visual mechanisms depends strongly on the spatiotemporal parameters of the visual patterns. But for coarse (i.e., low spatial frequency) and transient patterns it should be less than 100 ms (Georgeson, 1987; Watson, Ahumada, & Farrell, 1986). To ensure the low spatial frequency we fixed the nominal spatial frequency of the Gabor to 0.4 cycles/deg (we did not precisely control the viewing distance, so the actual spatial frequency varied somewhat between participants) and the size of the Gaussian window to 1.2 deg (2D standard deviation). The patterns had a vertical orientation. In the “fast” experiments (Experiments 2b and 2d), each cue was displayed for 10 ms and the delay between onset of two consecutive cues (the ISI) was 50 ms. To ensure that consecutive cues were processed independently by the visual system we (i) alternated the location on one side of the screen (e.g., ‘left-up’ and ‘left-down’) so that the smallest ISI at any one retinal location was 90 ms and (ii) alternated the phase of the patterns (90° and 270°).

Participants indicated their choice by pressing the left or right arrow keys on a keyboard. When the decision was correct, a money bag appeared on the chosen side. During the ITI, an animation displayed this money bag moving toward the bottom of the screen. When the decision was incorrect, no money bag appeared. All money bags collected by the participant remained at the bottom of the screen, so participants could track the amount of reward they had gathered during the current game.

The structure of the experiment was the same as Experiment 1, with the experiment consisting of a set of games of fixed durations and given difficulties. Each game consisted of a sequence of trials where participants could win a small reward if they made the correct decision or no reward if they made an incorrect decision. Games were again of three different types: (i) Type I, corresponding to easy games from Experiment 1, (ii) Type II corresponding to difficult games and (iii) Type 3 corresponding to mixed games. The type of the game was indicated by the color of the fixation cross – Type I: Green, Type II: Red and Type 3: Blue. The order of games was counterbalanced across participants.

In all four experiments the *up-probability* for easy and difficult games was 0.50 ± 0.22 and 0.50 ± 0, respectively. During mixed games, easy and difficult trials were equally likely. The reward rate-optimal policy for easy games was again to maintain constant threshold (Figure 2d) while for difficult it was to guess immediately (Figure 2e). Similarly, the optimal policy for the mixed condition was to start with a high boundary (similar to the boundary at the start of easy games) and steadily decrease it, eventually making a decision at *x* = 0 (Figure 2f). Just like in Experiment 1, participants were given training on each type of game and the reward structure was divided into three components: (approx $8.50) for participating, (approx 2 cents) for each correct response and (approx $25) for the participant accumulating the largest number of money bags.

### Results

We analyzed data using the same method as Experiment 1 after removing the nondecision time. *Wait* and *go* actions were used to determine the probability of *go*ing at all combinations of evidence and time, which were then used to determine a line of indifference, where the probability of wait matched the probability of go. We compared the slopes of this line of indifference for easy and mixed games for each of the four experiments.

#### Experiment 2a

**Figure 8 fig8:**
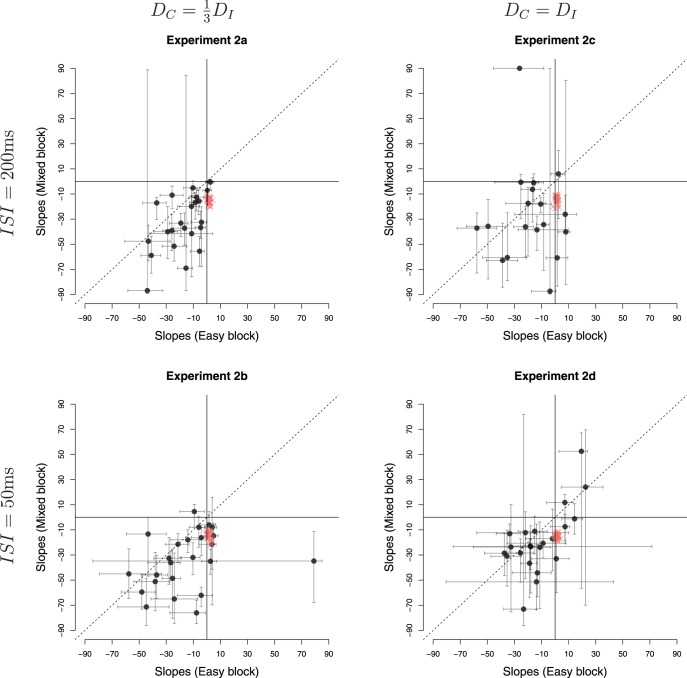
Slopes (in degrees) of estimated lines of indifference in easy versus mixed games in four experiments. The dashed line shows the curve for equal slope in easy and mixed games. Each circle (black) shows the estimated slopes for one participant. Error bars show 0.95 percentile bootstrapped confidence intervals. Crosses (red) show the estimated slopes for 24 simulated participants—decisions were simulated using a rise-to-threshold model with boundaries given by the optimal policies computed as described by (Malhotra et al., 2017). See the online article for the color version of this figure.

This experiment used the same parameters as Experiment 1, but replaced the Pacman game, with the evidence-foraging game described in Figure 7. Two participants failed the binomial test in the mixed games and were excluded from analysis. The mean RTs during easy, difficult and mixed games were 1155 ms (5.8 samples, *SEM* = 17 ms), 618 ms (3.1 samples, *SEM* = 27 ms) and 1142 ms (5.7 samples, *SEM* = 19 ms), respectively. We estimated the average number of stimuli that fell in the nondecision period (*ND*) to be 0.96, that is, a average nondecision delay of approximately 192 ms. For 21 out of the 22 participants, we estimated *ND* = 1 and for one participant no stimuli were excluded. Figure 8 (top-left panel) shows a comparison of the estimated slopes of lines of indifference in easy and mixed games. We observed that slopes were negative in both easy and mixed games for almost all participants and more negative during mixed games than easy games (*t*(21) = 3.92, *p* < 0.001, *m* = 15.76, *d* = 0.84). Again, circles show the slopes for estimated lines of indifference for each subjects. The 0.95 percentile confidence intervals on these slopes are obtained using the same bootstrap procedure described in Experiment 1.^6^ Note that the mean difference in slopes is virtually identical to Experiment 1, although the effect size (Cohen’s *d*) was larger during Experiment 1. Thus, Experiment 2a replicated the results of Experiment 1 showing that the findings were robust to different formulations of the evidence-foraging game. A model comparison exercise concurred with these results, showing that the majority of participants in mixed (*N* = 18) as well as easy games (*N* = 17) were better accounted by a logistic regression model using both evidence and time as a predictor than by a simpler model that used only evidence as the predictor (see Appendix D for details).[Fig-anchor fig8]

#### Experiment 2b

In the next experiment, we decreased the ISI to 50 ms and scaled the ITIs accordingly. All other parameters were the same as Experiment 2a. All participants passed the binomial test in the easy and mixed games. The mean RT during easy, difficult and mixed games were 337 ms (6.7 samples, *SEM* = 4.2 ms) 418 ms (8.4 samples, *SEM* = 9.8 ms) and 419 ms (8.4 samples, *SEM* = 5.3 ms), respectively, showing that this paradigm successfully elicited subsecond RTs typically found in signal detection paradigms. We estimated the average number of stimuli that fell in the nondecision period to be 3.7, that is, an average nondecision delay of approximately 183 ms. We estimated *ND* = 4 for 15 participants, *ND* = 3 for six participants, *ND* = 5 for two participants and *ND* = 1 for one participant. The bottom-left panel of Figure 8 shows the estimated slopes in easy versus mixed games. Like Experiment 2a, the slopes were negative for most participants in both easy and mixed games. Similarly, we also observed that the slopes were more negative in the mixed games than in the easy games, although the result was a little weaker than in Experiment 2a (*t*(23) = 2.12, *p* = 0.044, *m* = 11.80, *d* = 0.47). There are three possible reasons for this weaker result. First, the distribution for difference in slopes is more diffuse due the outlier at the right of the plot. Excluding this participant gave a clearer difference in slopes (*t*(22) = 3.31, *p* = .003, *m* = 15.19, *d* = 0.72) that was numerically highly similar to the slope difference observed in Experiments 1 and 2a. Second, for reasons discussed below, our estimates of nondecision time are likely to be less accurate in the “faster” paradigm. In turn, this error introduces variability in the accuracy of the actual evidence paths on a trial-by-trial basis that were used to derive our slope estimates. Lastly, it is possible that the process decreasing the boundary needs time to estimate the drift and adjust the boundary accordingly. With shorter ISI, this process may have less time to affect the decision process before the response is made, resulting in smaller difference in slopes between conditions.

#### Experiment 2c

Next, we changed the ISI back to 200 ms (same as Experiment 2a) but increased *D*_*C*_, the ITI for correct decisions, to the same value as *D*_*I*_, the ITI for incorrect decisions (10s for both). Increasing the ITI decreased the reward per unit time and meant that participants had to wait longer between trials. Participants found this task difficult, we suspect because the ITI is so much longer than the typical RT. That is, participants spend most of their time waiting for a new trial, but then those trials are over rather quickly. Perhaps as a result, the games lacked in engagement and six out of 24 participants failed the binomial test in mixed games. For the 18 remaining participants, the mean RTs in easy, difficult and mixed games were 807 ms (4.0 samples, *SEM* = 27 ms), 858 ms (4.3 samples, *SEM* = 49 ms) and 954 ms (4.8 samples, *SEM* = 32 ms), respectively. We estimated the average number of stimuli that fell in the nondecision period to be 0.9, that is, an average nondecision delay of approximately 176 ms (*ND* = 2 for 21 participants and *ND* = 0 for the remaining three participants). The estimated slopes are shown in the top-right panel of Figure 8. We observed much greater variability in the estimated decision boundaries, though slopes were generally negative in mixed as well as easy games.^7^ The mean estimated slopes decreased more rapidly in mixed games as compared to easy games. However, given the large variability of responses and the number of participants that had to be excluded, this effect was comparatively weaker (*t*(17) = 2.41, *p* = .028, *m* = 17.97, *d* = 0.60). Nevertheless, the mean slope difference is very similar to that observed in all previous three experiments.

#### Experiment 2d

In this experiment we tested the final permutation of ISIs and ITIs—we decreased the ISI to 50 ms and matched the ITIs for correct and incorrect decisions (both 2.5s). Two participants failed the binomial test in mixed games and were excluded from further analysis. The mean RTs during easy, difficult and mixed games were 240 ms (4.8 samples, *SEM* = 4 ms), 308 ms (6.2 samples, *SEM* = 9 ms) and 294 ms (5.9 samples, *SEM* = 5 ms), respectively. We estimated the average number of stimuli that fell in the nondecision period to be 4.3, that is, an average nondecision delay of approximately 216 ms (*ND* = 4 for 18 participants, *ND* = 5 for five participants and *ND* = 7 for one participant). The bottom-right panel in Figure 8 compares the estimated slopes in easy and mixed games. The mean slope in either kind of game was negative (*t*(21) = −3.10, *p* = .005, *m* = −11.66 for easy games and *t*(21) = −3.42, *p* = .003, *m* = −18.82 for mixed games). However, in contrast to Experiment 2b, there was no significant difference in mean estimated slopes during easy and mixed games (*t*(21) = 1.71, *p* = 0.10, *m* = 7.15, *d* = 0.32).

### Discussion

Experiments 2a-d revealed three key behavioral patterns: (i) participants generally decreased their decision boundaries with time, not only in the mixed games, but also in the easy games, (ii) this pattern held for the rapid task (Experiment 2b) but the variability of parameter estimates increased at faster RTs, (iii) decreasing the difference between *D*_*C*_ and *D*_*I*_ decreased the difference in slopes between easy and mixed games.

**Figure 9 fig9:**
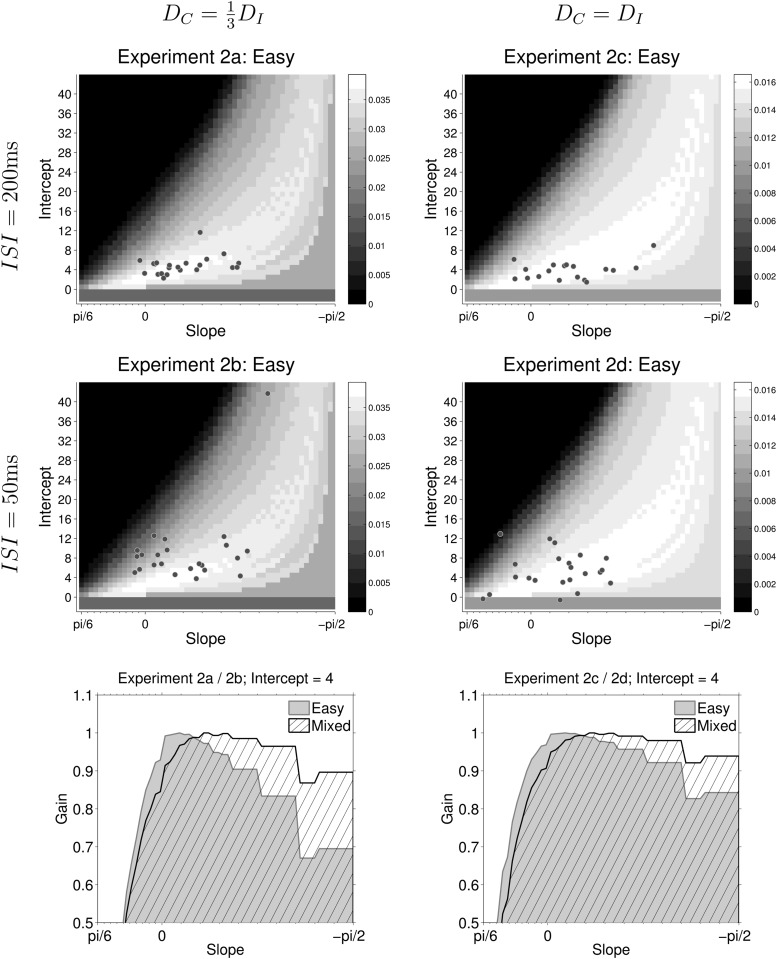
Reward rate for Experiments 2a through 2d. Each heat-map in the first two rows shows the “landscape” of reward rate in policy space and dots show estimated policies adopted by participants in easy games. The bottom row show profiles sliced through the (normalized) reward-rate landscape at a particular intercept. Shaded regions show profiles for easy games, whereas hatched regions show profiles for mixed games.

Clearly, it is not optimal to decrease the decision boundary during fixed difficulty (easy) games, but most participants seemed to do this. As noted in Experiment 1, a possible reason is that the reward rate for suboptimal policies is asymmetrical around the optimal boundary. Figure 9(a) shows the reward rate landscape for all possible decision boundaries during easy games in Experiment 2a, and maps the estimated boundaries for each participant onto this landscape.[Fig-anchor fig9]

Reward rate is maximum at (0, 3). When slope increases above zero the reward rate drops rapidly. In contrast, when slope decreases below zero, reward rate decreases gradually. This asymmetry means that participants pay a large penalty for a suboptimal boundary with a positive slope, but a small penalty for a suboptimal boundary with a negative slope. If participants are uncertain about the evidence gathered during a trial, or about the optimal policy, it is rational for them to decrease their decision boundary, as an error in estimation will lead to a relatively small penalty. Figure 9 suggests that most participants err on the side of caution and adopt policies with high (though not maximum) rewards and decreasing boundaries.

The shape of the reward landscape also sheds light on why participants behave differently when the ITI *D*_*C*_ is changed, even though changing this parameter does not affect the optimal policy. The first column in Figure 9 shows the reward rate in experiments where $D_{C}=\frac{1}{3}D_{I}$, while the second column shows the reward rate in experiments where *D*_*C*_ = *D*_*I*_. The top two rows show the reward-rate landscapes in easy games at all combinations of slopes and intercepts, while the bottom row compares the reward rate in easy and mixed games at a particular intercept of decision boundary but different values of slope (i.e., a horizontal slice through the heat-maps above). Even though the optimal policy in all four experiments is the same, there are several ways in which the reward-rate landscape in the left-hand column (Experiments 2a and 2b) differ from the landscape in the right-hand column (Experiments 2c and 2d).

First, the reward-rate landscape in easy games is more sharply peaked when $D_{C}=\frac{1}{3}D_{I}$ (Experiments 2a and 2b). This is most clearly discernible in panels in the bottom row which shows the profile of the (normalized) reward-rate landscape at a particular intercept. If the participant adopts a boundary with large negative slope, the difference between the reward rate for such a policy and the optimal reward rate is larger when $D_{C}=\frac{1}{3}D_{I}$ (left panel) than when *D*_*C*_ = *D*_*I*_ (right panel). So in Experiments 2a and 2b adopting a suboptimal policy carries a larger ‘regret’ than in Experiments 2c and 2d. This means that the reward landscape constrains the choice of boundaries more in Experiments 2a and 2b than it does in Experiments 2c and 2d, even though the optimal policy for all experiments is the same.

The panels in the bottom row also compare the reward-rate profiles during easy (shaded) and mixed (hatched) games at a particular intercept. It can be seen that for both types of experiments the normalized reward rate is larger in mixed games than easy games when slopes are more negative. Thus it is better (more rewarding) to have decreasing boundaries in mixed games than in easy games. However, the difference in easy and mixed games is larger when $D_{C}=\frac{1}{3}D_{I}$ (left panel) than when *D*_*C*_ = *D*_*I*_ (right panel). Correspondingly, we found a more robust difference in slopes during Experiments 2a and 2b than we did in Experiments 2c and 2d.

The third behavioral pattern was an increase in variability of slopes when the decisions were made more rapidly. There are two possible sources of this variability: internal noise and error in estimation of the nondecision time. Recall that we excluded stimuli that arrive during the nondecision time based on a single estimate of this time for each participant. It is likely that the nondecision time varies from trial-to-trial; indeed, this is a common assumption in models of decision making (see Ratcliff & Smith, 2004). Any such variability means that on some trials we are including irrelevant samples (estimating a nondecision time too short) or excluding relevant samples (estimating a nondecision time too long). As a result, there is a discrepancy between the evidence paths that actually led to the participant’s decision and the one entered into the logistic regression model used to estimate the decision boundary. Importantly, this discrepancy will be much smaller in the experiments with a long ISI, because even an error in nondecision time of, say, 100 ms will at most introduce only one additional or excluded evidence sample. However, in the experiments with a much shorter ISIs, the same numerical error will result in several additional or missed evidence samples. Therefore, trial-to-trial variability in the nondecision times introduces more noise in the slope estimates for the faster experiments.

## Experiment 3

In the above experiments, the optimal policy was to decrease decision boundaries in mixed games but keep them constant in single-difficultly games. Correspondingly, data suggested that participants adopted more strongly decreasing boundaries in mixed-difficulty games than in single-difficultly games, particularly when errors are costly (in terms of reward rate). In Experiment 3 we changed the parameters so that the optimal policy during mixed-difficulty games was, in fact, to increase the decision boundary. Recall from the theory on optimal shapes of decision boundaries that the optimal policy in mixed games is to decrease decision boundaries only when one of the decision types is extremely difficult. In contrast, when both types of decisions are easy or moderately difficult, the policy that optimizes reward rate is to increase decision boundaries or leave them constant (Figure 2c). Therefore, if participants were optimizing their average reward, we expected estimated slopes in mixed-difficulty games of this type to be either the same or larger than slopes in single-difficultly games.

Experiment 3 used the same experimental paradigm as Experiment 2. The parameters for Experiment 3 are shown in Table 3. During this experiment, easy games showed cues with *up-probability* $\frac{1}{2}\pm 0.40$ – so participants could make really rapid decisions in these games. And unlike Experiments 1 and 2, difficult games showed cues with *up-probability* $\frac{1}{2}\pm 0.10$. The optimal boundaries in this case are higher for difficult games than easy games (Figures 2a and 2b) and the optimal boundary for mixed games show a slight increase in evidence with time (Figure 2c).[Table-anchor tbl3]

Twenty-four participants played blocks of easy, difficult and mixed games with the objective of maximizing their reward. Each correct decision was worth 2p and there was no reward or penalty for incorrect decisions. The participant who collected the largest number of money bags received a bonus reward of (approx $25). The ISI was 50 ms and ITI was 3.5 s.

**Figure 10 fig10:**
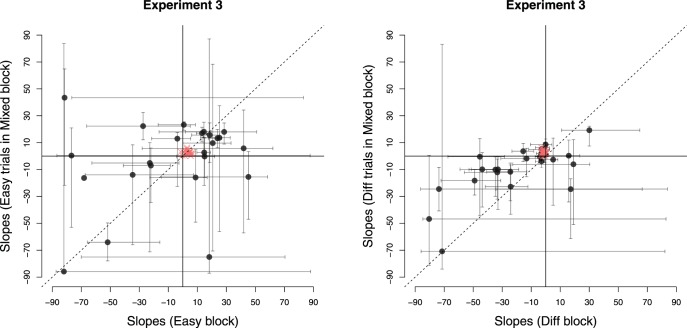
Slopes of estimated lines of indifference in Experiment 3. The panel on the left compares slopes for the easy trials during mixed games with slopes for all trials during easy games, whereas the panel on the right compares slopes for the difficult trials in mixed games with slopes for all trials during difficult games. In each panel, the solid vertical and horizontal lines show lines of zero slope (flat threshold), and the dashed line shows the curve for equal slopes in the two types of games. Each circle shows the estimated slopes for one participant and crosses show estimated slopes from simulated optimal participants. See the online article for the color version of this figure.

We used the same procedure as the above experiments to analyze the data. All participants passed the binomial test in mixed games so no data was rejected. Figure 10 shows the estimated slopes for lines of indifference in easy, difficult and mixed games. Unlike the previous experiments, we compared the slopes in mixed games not only to easy games, but also to difficult games, since the difficult games in this case required participants to accumulate evidence before making a decision.[Fig-anchor fig10]

During easy games, participants made really rapid (and accurate) decisions, with mean RTs 107 ms (*SEM* = 4 ms), that is, based on two to three sample cues (after excluding nondecision time). Such fast responses are of course consistent with the model prediction of narrow decision boundaries in this condition. As discussed above, on this rapid time scale noise in the responses due to nondecision time or due to variability of the perceptual system has a large impact on the variability of estimated slopes. Indeed, we can see from Figure 10 (left panel), that the confidence intervals around estimated slopes are large and there was substantial (between-participants) variability in the mean estimated slopes.

A more accurate comparison between single- and mixed-difficulty games is obtained by comparing the slopes in difficult games with the slopes on difficult trials in mixed games. The panel on the right in Figure 10 shows this comparison. The mean RTs in difficult games was 397 ms (8 samples, *SEM* = 9 ms), while that in the mixed games was 234 ms (4.7 samples, *SEM* = 6 ms). We estimated the nondecision time to be approximately 4.5 samples, that is, 227 ms (*ND* = 5 for 13 participants and *ND* = 4 for the remaining 11 participants). Like previous experiments, the mean slope in single-difficultly (here, difficult) games was less than zero (*t*(23) = −3.15, *p* < 0.001, *m* = −19.5).

Crucially, in contrast to Experiment 1 and 2, but in agreement with the reward-rate optimizing policy, we found that the estimated slopes in mixed games were slightly *larger* (less negative) than in the difficult games (*t*(23) = −2.25, *p* = .034, *m* = −9.86, *d* = −0.39). Indeed, model comparison suggested that, again in contrast to Experiment 1 and 2, the simpler logistic regression model using only evidence as the predictor provided a better account of data in mixed games than the model using both evidence and time as predictors (see Appendix D). However, for the difficult games, the evidence was more mixed in that for just over half the participants, a model that included time as a predictor performed better. These results are consistent with the slope comparisons in that boundaries varied with time (slightly) in difficult games, but were approximately constant in the mixed games. Although we do not actually observe *increasing* boundaries, the shift from decreasing to approximately constant boundaries is a shift in the right direction.

**Figure 11 fig11:**
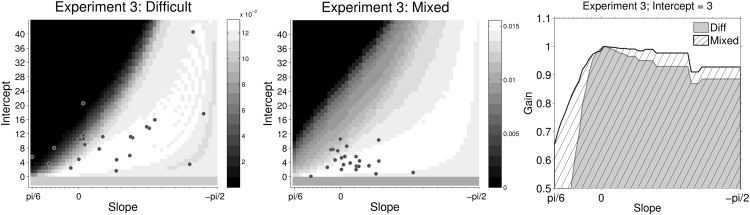
Reward rate during difficult and mixed games during Experiment 3. Each heat-map shows the “landscape” of reward per unit time in policy space. Lighter colors show higher reward. Each circle shows the slope and intercept of the estimated line of indifference for a participant. The right-most panel compares the reward landscape in difficult and mixed games at a particular intercept.

In Figure 11, we have again plotted the estimated policies of all participants on the reward landscape. The key difference in behavior between mixed and difficult games was that most participants were concentrated around the zero slope during mixed games while the slope of boundaries chosen by participants in the difficult games were spread over a large range with a number of participants choosing policies with large negative slopes. In the right-most panel, we have compared the profile of the landscape, slicing it at intercept = 3 (optimal policy in mixed games had slope slightly above 0 and intercept between 3 and 4). This profile shows that, like Experiment 2, reward rate is an asymmetric function of slope in both the difficult and mixed games. However, in contrast to Experiment 2, the amount of asymmetry is now lesser during mixed games than difficult games. So during mixed games, participants can choose policies in the neighborhood of constant boundary with a lower regret, even in the presence of uncertainty about the evidence or the optimal boundary. This could explain why the majority of participants in the mixed games are concentrated around policies with zero slope. In contrast, the larger asymmetry during difficult games seems to push a number of participants into adopting boundaries with large negative slopes—a lower risk strategy that nevertheless leads to a small loss in average reward.[Fig-anchor fig11]

## General Discussion

### Constant or Decreasing Boundaries

Sequential sampling models have had a very successful history of fitting data in a variety of decision-making experiments (Bogacz et al., 2006; Ratcliff, 1978; Ratcliff & Smith, 2004; Ratcliff et al., 2016; Smith & Vickers, 1989). These models typically assume that decision boundaries remain constant during a trial, so introducing the possibility of changing boundaries adds further complexity to these models. The question is whether this complexity is warranted given existing data.

Recently, Hawkins et al. (2015) and Voskuilen, Ratcliff, and Smith (2016) conducted a model comparison based on data from a number of decision-making studies and found that introducing decreasing bounds did not generally improve the model fit. In this study, we took a different approach—instead of working out whether decreasing boundaries improves model fit, we used a mathematical model (Malhotra et al., 2017) to establish the circumstances for changing decision boundary if the decision maker wanted to maximize reward rate. The key insight from this approach is that optimal decision boundaries decrease only in very specific scenarios—when one of the difficulty in a mixed-difficulty task is extremely difficult, or even impossible. In other conditions, optimal boundaries for mixed-difficulty tasks may increase or stay constant based on the difficulty of constituent decisions. An advantage of the model presented in this study is that it can be used for inferring the reward rate of any given boundary, which can then be used to compare with the optimal boundary. Using this approach, we found that suboptimal policies were “asymmetrically distributed” near the optimal boundary in policy space. A judicious decision maker should consider this asymmetry in reward landscape to make decisions that are robust to uncertainty in task parameters and to their own estimate of the optimal policy. Six expanded-judgment experiments indicate that people may not only be modulating how decision boundaries change with time, but may also be using such robustness considerations to choose the value and shape of these boundaries.

So why do Hawkins et al. (2015) and Voskuilen et al. (2016) find no strong evidence for changing decision boundaries and, indeed, why are models with constant decision thresholds so successful at fitting data from a variety of experiments? There could be three possible reasons. First, the data sets analyzed by Hawkins et al. (2015) and Voskuilen et al. (2016) consist of mixed-difficulty experiments with a variety of different difficulty levels. For example, Experiment 1 conducted by Hawkins et al. (2015) was a motion-discrimination task with six different difficulty levels (0%, 2.5%, 5%, 10%, 20% and 40%) while Experiment 1 from Ratcliff, Hasegawa, Hasegawa, Smith, and Segraves (2007) was a brightness-discrimination task with three levels of difficulty (55%, 65% and 98%). It is not clear in any of these experiments what the shape of boundaries that optimize reward rate should be. As we have discussed above, optimal boundaries do not necessarily decrease in mixed-difficulty trials and when they do decrease, the rate of decrease varies over a broad range based on the levels of difficulty. So even if participants were optimizing reward rate in the experiments considered by Hawkins et al. (2015) and Voskuilen et al. (2016), this may not necessarily entail observing decreasing boundaries.

Second, each of our experiments carefully controls the cost/reward of each decision and links performance to reward. This allows us to compute the optimal behavior in the task (in terms of reward rate) and compare participant performance with this optimal behavior. In contrast, most studies considered by Hawkins et al. (2015) do not have a performance-based reward structure. Participants are asked to emphasize speed, accuracy or both and there is no explicit scale on which a participant can measure the expected return of a policy. Exceptions to these are studies involving nonhuman primates, such as Roitman and Shadlen (2002); Ratcliff, Cherian, and Segraves (2003); Ditterich (2006), where performance was explicitly linked to reward and interestingly, Hawkins et al. find evidence for decreasing boundaries in these studies.

Decisions in ecologically realistic situations are typically accompanied by costs and rewards and the structure of incentives can profoundly affect performance, as shown by a series of studies in experimental economics (Camerer & Hogarth, 1999; Cubitt, Starmer, & Sugden, 1998). Therefore, if we want to establish whether participants decrease decision boundaries within a trial, we must determine what it is they stand to gain by changing their decision boundaries during the experiment.

Last, note that the expanded-judgment paradigm used by us is different from the signal detection paradigms used in studies analyzed by Hawkins et al. (2015) and Voskuilen et al. (2016). This is a key strength of our study as we are able to observe the exact sequence of stimuli observed by the decision maker and infer their decision boundaries based on these observations. It has been demonstrated recently that constraining sequential sampling models by the exact sequence of stimuli provides a closer description of RTs than that obtained from models in which the drift parameter is assumed constant within a trial (Park, Lueckmann, Kriegstein, von Bitzer, & Kiebel, 2016). However, using this paradigm leaves open the possibility that the decision boundary is set differently when the decision processes draw samples from an internal representation (e.g., in color/brightness/numerosity judgment tasks) and when samples drawn cannot be recorded by the experimenter. Previous evidence suggests that results from expanded-judgment tasks can be generalized to situations where sampling is internal (Vickers, Burt, et al., 1985; Vickers, Smith, Burt, & Brown, 1985). However, these studies did not examine a signal-detection task where RTs are typically <500 ms. Thus, an important outstanding question is whether people use different decision processes for internally and externally sampled observations and whether this affects how they set their decision boundaries.

Data from several expanded-judgment tasks involving choice between multiple alternatives have been successfully analyzed using sequential sampling models with fixed boundaries, which have been shown to capture the key interesting aspects of these data (Brown, Steyvers, & Wagenmakers, 2009; Hawkins, Brown, Steyvers, & Wagenmakers, 2012). It would be interesting to extend the model fitting methodology presented in this paper to the case of choice between multiple alternatives, and investigate if these data are better described by a model with flat or decreasing boundaries.

### Individual Differences

In all of the preceding experiments, we observed variability in behavior both between individuals and between trials within a participant. We have already discussed two reasons for the variability between trials: (a) nondecision time, which is estimated per individual but may vary from trial-to-trial and (b) internal noise, which could lead to a trial-to-trial variability in drift rate. As mentioned previously, a trial-to-trial variability in drift rate, starting point or threshold has been shown to be essential for fitting RT distributions—in particular, different patterns of error RTs—using sequential sampling models (see Ratcliff, 1978; Ratcliff & Smith, 2004). In addition to these, our study highlights another source of variability between individuals—the shape of the reward landscape with its broad region in which acceptably high reward rates could be achieved. Reward rate was asymmetrically distributed around the optimal policy in all the above experiments, with a bias toward suboptimal policies that yielded a reward rate that was close to maximum.

A number of previous studies have compared individuals with optimal behavior in decision-making tasks and found that participants often use boundaries that are suboptimal (Bogacz, Hu, et al., 2010; Pitz et al., 1969; Sanders & Linden, 1967; Simen et al., 2009; Starns & Ratcliff, 2010; Wallsten, 1968; Zacksenhouse, Bogacz, & Holmes, 2010). It has also been observed that participants have a tendency to overvalue accuracy, setting boundaries that are wider than those suggested by maximization of reward rate (Balci et al., 2011; Bohil & Maddox, 2003; Maddox & Bohil, 1998; Myung & Busemeyer, 1989; Starns & Ratcliff, 2012). To explain this behavior, a set of studies have investigated alternative objective functions (Bogacz et al., 2006; Bohil & Maddox, 2003; Zacksenhouse et al., 2010). For example, Zacksenhouse et al. (2010) found that only about 30% of participants achieve (reward rate) optimality and the behavior of the other 70% is better explained by a robust strategy that maximizes performance under presumed level of uncertainty (the maximin strategy).

The behavior of participants in our experiments is in line with such a robust strategy: a small proportion of participants adopt policies that are close to optimal (Figures 5, 8, and 10) but most participants adopt strategies that yield high, but not maximum, reward rate (Figures 6, 9 and 11). Because the gradient of reward rate was larger above constant boundary than below it, this meant choosing a policy with a decreasing boundary.

In the preceding experiments, there can be several sources of uncertainty, leading to adoption of a robust strategy: uncertainty in estimation of task parameters such as ISI/ITI, uncertainty in the signal due to noise in the sensory system, and uncertainty in the estimate of reward rate for the task. If participants use a hill-climbing learning mechanism (Myung & Busemeyer, 1989; Simen, Cohen, & Holmes, 2006), these uncertainties introduce noise in the learning process and make it harder for participants to search for the optimal policy, especially when the reward landscape has a low gradient, leading to the observed differences in the choice of boundaries. With training, participants should be able to reduce these uncertainties and approach optimal boundaries, as shown by previous research (Myung & Busemeyer, 1989; Balci et al., 2011).

Conversely, when internal noise in the sensory system increases or when the estimate of the task parameters becomes more uncertain, participants should find it more difficult to locate the optimal policy in policy space. For example, it has been shown that the duration estimates of older adults are more variable than younger adults (Block, Zakay, & Hancock, 1998) and visual perception declines with aging (Habak & Faubert, 2000; Owsley, 2011; Spear, 1993; Weale, 1963). These processes will increase the level of uncertainty in the (temporal) task parameters as well as the visual stimuli and could explain why older adults adopt boundaries that are farther from optimal (Starns & Ratcliff, 2010, 2012). Of course, it is also possible that the deviation from optimality is a consequence of not only an increase in visual and temporal noise but also a decline in the ability to flexibly set the boundary and more empirical studies would be required to tease apart the relative contribution of these two factors.

### Mechanistic Considerations

The behavior of participants in the experiments above suggests that they adapt their decision-making mechanism to achieve near-maximal reward rates. We claim neither that participants are optimal—they clearly are not—nor that the mathematical model we have used to derive the optimal policy is a psychological theory. The focus of our study was not on establishing the mechanism by which people achieve this behavior but on comparing the normative behavior with the empirical behavior. In a manner similar to “ideal observer models” in the study of sensory systems (Geisler, 2003), specifying the optimal policy has helped us (a) identify experimental conditions that are best suited to empirically test time-varying decision boundaries, and (b) identify sources for suboptimal behavior (or inefficiencies) through analysis of the reward landscape. Nevertheless, we finish with some considerations of the underlying mechanisms that may be responsible for the time varying boundaries observed in our study.

First of all, the reader may wonder whether the decreasing bounds we identify in our experiments may be accounted for by existing mechanisms in models that assume constant boundaries. In Appendix C, we explore two such mechanisms—between-trial noise in the drift rate and imperfect integration of information. We simulated decisions using a rise-to-threshold model both with and without between-trial noise in drift rate and with and without loss in integration of evidence. We then estimated the slopes of boundaries using the method discussed above and found that the estimated difference in slopes between single- and mixed-difficulty conditions reflected the true difference, irrespective of the noise in drift rate or loss in integration of evidence. Thus, our inferences about difference in slopes remain valid even when these mechanisms are considered.

Next, the pattern of decision making we observed in the expanded-judgment tasks is compatible with a number of different mechanistic accounts. For example, it is possible that participants did not weigh each cue equally and cues later in the decision carried a larger weight. This mechanism has been recently suggested by the urgency-gating model (Cisek, Puskas, & El-Murr, 2009; Thura, Beauregard-Racine, Fradet, & Cisek, 2012). Similarly, it is also possible that participants maintained a constant threshold but also used a stochastic deadline. That is, they maintain an internal clock and make a decision if evidence crosses a constant threshold before a deadline or choose the most-likely alternative if the threshold is not crossed but a deadline is reached. This mechanism is similar to the response signal paradigm (e.g., Ratcliff, 2006), with an internal instead of an external deadline. Both these mechanisms will lead to decision boundaries that appear to decrease with time. However, the urgency gating model does not assume integration of sensory input over whole duration of trial, but rather rapid forgetting of previously integrated input. It would be interesting to formally compare in a future study whether the urgency gating model or an integration to boundary model better describes data from the current study, which is freely available, as mentioned earlier.

**Figure D1 fig16:**
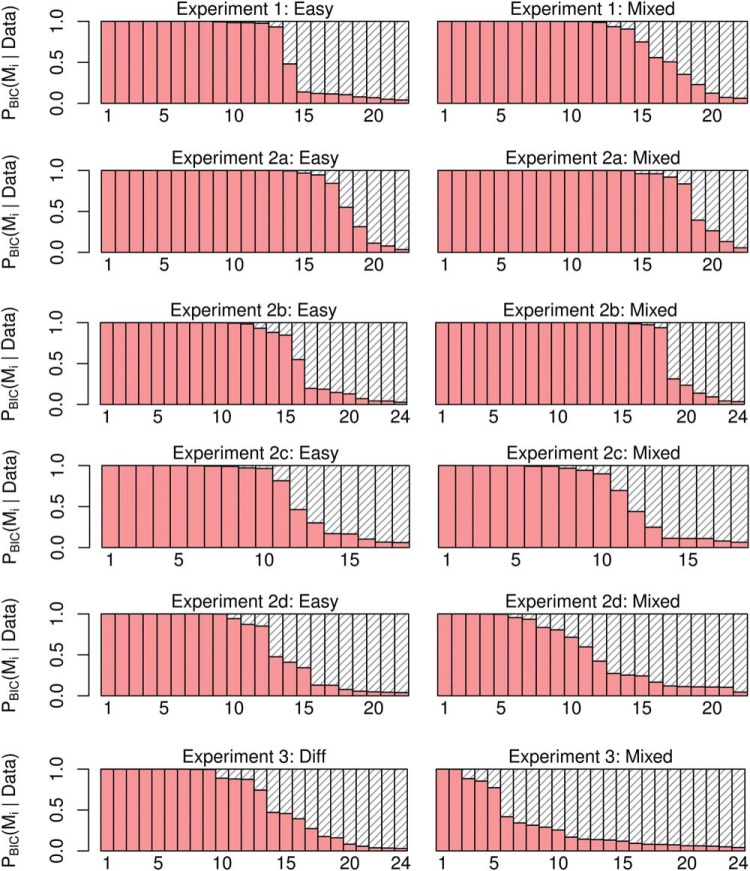
Each row shows the posterior model probability for the logistic regression models using only evidence as the predictor (hatched) and using both time and evidence as predictors (shaded) for all participants in an experiment. The left and right-hand columns show these posterior probabilities during the single- and mixed-difficulty conditions, respectively. See the online article for the color version of this figure.

However, note that the normative model does not always predict that decision boundaries should decrease with time. In agreement with this, we found that many participants in Experiment 3 did not appear to decrease their decision boundaries in mixed-difficulty condition (also see Figure D1). These findings are not straightforward to reconcile in mechanistic accounts such as urgency-gating and stochastic deadline and provide a good test for teasing apart these models.

The logistic regression model used to infer the boundary from data (Equations 2 and 3) assumes that people integrate evidence to a constant boundary but that the slope of the boundary is allowed to vary. Under this assumption, participants appear to decrease their decision boundaries when decreasing boundaries increases reward rate. So the thrust of our argument is that people seem sensitive to the normative behavior and when the normative behavior changes (single-difficulty vs. mixed-difficulty conditions) participants seem to adapt their decision mechanism in line with the normative standard. A separate and important question is how people make this adaptation. Decreasing the decision boundary, increasing the gain of observations or maintaining a stochastic deadline are all possible mechanisms to achieve this goal and future research should examine what mechanisms are used by people.

## Figures and Tables

**Table 1 tbl1:** Values of Parameters Used During the Game

Parameter name	Value
Interstimulus interval (ISI)	200 ms
Intertrial interval (ITI), correct (*ISI* × *D_C_*)	3 s
ITI, incorrect (*ISI* × *D_I_*)	10 s
Reward	(approx 2 cents)
Drift for easy condition (ϵ_*e*_)	.20
Drift for difficult condition (ϵ_*d*_)	0
Block duration, training	150 s
Block duration, testing, easy	240 s
Block duration, testing, difficult	300 s
Block duration, testing, mixed	300 s

**Table 2 tbl2:** Values of Parameters for Experiment 2

Parameter name		Value
Drift for easy condition (ϵ_*e*_)		.22
Drift for difficult condition (ϵ_*d*_)		0
Reward		(approx 2 cents)
	*D_I_* = *ISI*×50	
ISI	$D_{C}=\frac{1}{3}D_{I}$	*D*_*C*_ = *D*_*I*_
200 msec	Experiment 2a	Experiment 2c
50 msec	Experiment 2b	Experiment 2d
*Note*. The parameters that are common to all four subexperiments are listed at the top. Each of the four subexperiments has a different combination of interstimulus and intertrial intervals, the values of which are listed at the bottom. ISI = interstimulus interval.		

**Table 3 tbl3:** Values of Parameters Used During Experiment 3

Parameter name	Value
Drift for easy condition (ϵ_*e*_)	.40
Drift for difficult condition (ϵ_*d*_)	.10
Reward	(approx 2 cents)
Intertrial interval, correct	3.5 s
Intertrial interval, incorrect	3.5 s
Interstimulus interval	50 ms

**Figure B1 fig13:**
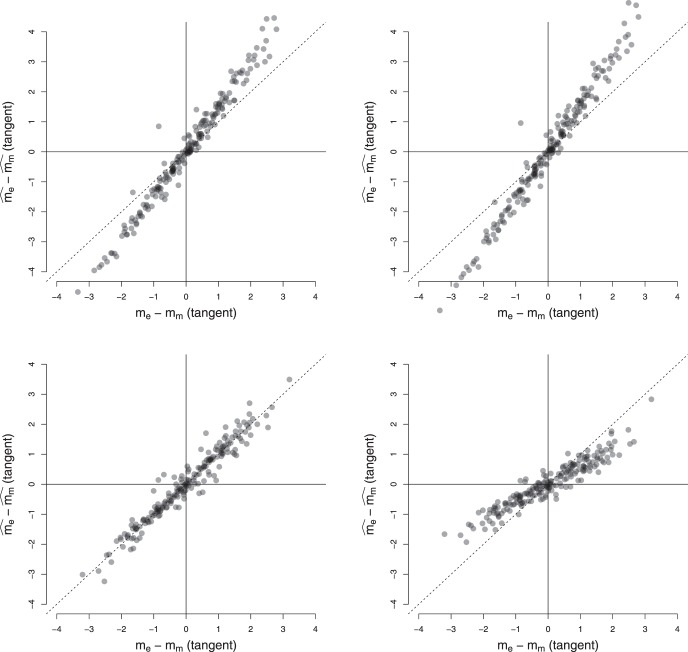
True versus estimated difference in slopes for 200 simulations. Each circle represents one participant simulated using either a rise-to-threshold model (first row) or a probabilistic boundary model (second row). The figures in the left column compare true difference with the difference estimated using logistic regression (as described in Section ‘Experiment 1’), whereas the panels in the right column make the same comparison but slopes are estimated by fitting a rise-to-threshold model.

**Figure C1 fig14:**
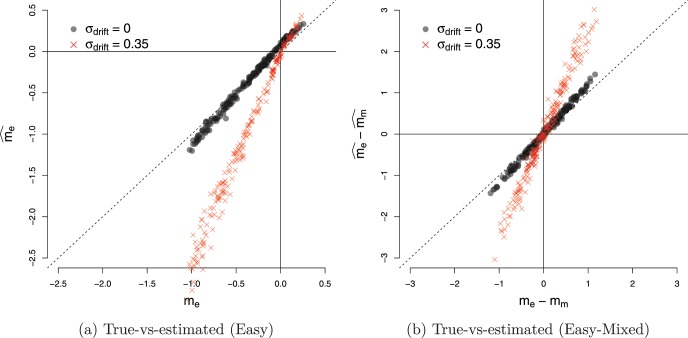
The effect of trial-to-trial variability in drift on estimation of slopes of decision boundary. Panel (a) shows a comparison of true and estimated slopes in a single-difficulty task (ϵ = 0.22). Panel (b) shows a comparison of true and estimated difference in slope during an easy (ϵ = 0.22) and mixed (ϵ = 0.22/0) task. In both panels, each dot represents a participant simulated using a rise-to-threshold model with no trial-to-trial drift and crosses represent participants simulated using a rise-to-threshold model with a large trial-to-trial variability in drift (σ_*drift*_ = 0.35). See the online article for the color version of this figure.

**Figure C2 fig15:**
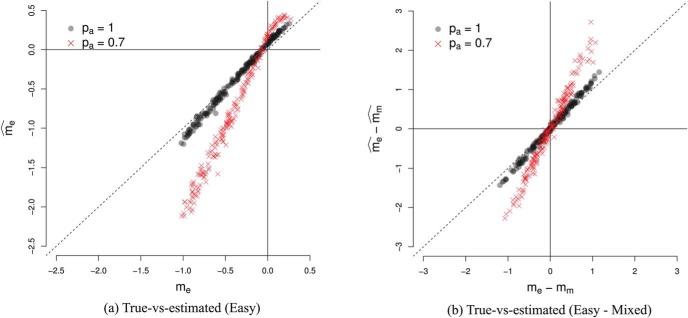
The effect of information loss on estimation of slopes of decision boundary. Panel (a) shows a comparison of true and estimated slopes in a Single-difficulty task (ϵ = 0.22). Panel (b) shows a comparison of true and estimated difference in slope during an Easy (ϵ = 0.22) and Mixed (ϵ = 0.22/0) task. In both panels, each dot represents a participant simulated using a rise-to-threshold model without any information loss and crosses represent a participant simulated using a binomial loss model (*p_a_* = 0.7). See the online article for the color version of this figure.

**Figure D2 fig17:**
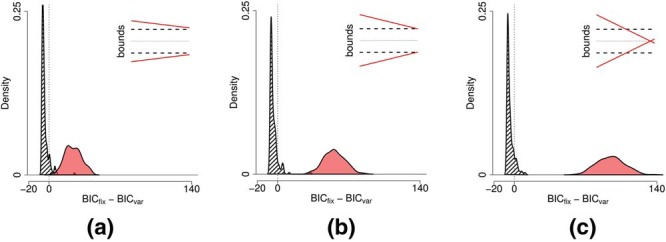
Each plot shows the distribution of difference in Bayesian information criterion (BIC) values for fixed boundary model (only evidence used as predictor) and variable boundary model (both evidence and time used as predictors). Hatched distributions show this difference in BIC values when decisions are simulated using integration of evidence to a fixed boundary (inset, dashed line) and shaded distributions show the difference in BIC values when decisions are simulated from integration to a decreasing boundary (inset, solid line). Three different slopes are used for decreasing boundaries (a) 15 degrees, (b) 30 degrees, and (c) 60 degrees. See the online article for the color version of this figure.

## Footnotes

1 Article available at https://osf.io/9t76q/

2 See, for example, Palmer et al. (2005) who tested 6 participants with ∼560 trials for each participant and Ratcliff and McKoon (2008) who tested 15 participants with ∼960 trials per participant. We used a performance-based reward paradigm (outlined subsequently), which means that the number of trials varied between participants and experiments but were between ∼150 and ∼650 trials per participant for each of the experiments reported subsequently.

3 Each confidence interval is based on percentiles of the bootstrap distribution of the lines of indifference. Each bootstrap distribution is obtained by generating 1,000 independent bootstrapped data sets (per condition) and computing the slope for the line of indifference on the data set. Each data set consists of *N* sampled trials, where *N* is the number of trials (for that condition) seen by the participant.

4 Analogous analysis was also done for Experiments 2a through 2d as well and no significant differences were found.

5 The luminance of the monitor was gamma-corrected so that luminance was a linear function of grayscale RGB value. The background luminance was fixed to 0.5 on a scale of [0, 1].

6 When the estimated slope for a participant is really steep, a large negative slope is qualitatively similar to a large positive slope. For this reason, the confidence intervals for some participants with large slopes seem extremely wide. We compare the slopes using a circular difference (Equation 4), which corrects for this problem.

7 There is an outlier who seems to have a large positive slope in mixed games. This may seem unintuitive, but, as noted above, there is little difference between a large positive and a large negative slope—in both cases the probability of going depends strongly on time (a temporal deadline) and weakly on evidence. The circular difference (Equation 4) accounts for cases like this.

## A Nondecision Time

Figure A1 illustrates the reverse correlation procedure used to compute nondecision time across the experiments. For this example participant, the proportion of easy-difficulty trials where the samples at time steps (1, 2, 3, 4, 5, 6, . . .) before the response were in the same direction as the responses were (0.62, 0.65, 0.63, 0.68, 0.85, 0.91, . . .). The first time-step where this proportion was larger than 0.75 was 5, and we estimated the nondecision time to be 4. Note that the correlation during the nondecision time is not 0.50 because of drift in the stimuli—the responses made by participants are generally correlated to the stimulus (provided their response rates were better than chance, which we check for in our exclusion criterion). Hence it is important to pick a threshold that is larger than the drift. We experimented with a number of different threshold values. Results remained similar for a range of threshold values that were above the drift rate but below the highest correlated stimuli for the participant. The threshold value chosen in this manner was kept constant across participants and experiments.[Fig-anchor fig12]

Note that this analysis method assumes a fixed nondecision time across trials. Because nondecision time would, presumably, vary from trial to trial, we carried out simulations to test how trial-by-trial variability in nondecision time affects our estimates of the slopes of the decision boundaries. We found that the analysis method outlined in the main text was robust under trial-to-trial variability in nondecision time: Adding a trial-by-trial variability added a small amount of noise in our estimates but estimated slopes were still highly correlated with true slopes and inferences about difference in slopes between conditions remained the same irrespective of the variability of nondecision time.

## B Parameter Recovery

In this study, we estimated decision boundaries based on a line of indifference computed using a logistic regression model. We now show that this estimation method allows us to make valid comparisons about slopes of single- and mixed-difficulty games and that our inferences about difference in slopes are valid irrespective of whether the noise in decisions originates from stochasticity in *wait–go* actions (as we assume) or from a noisy integration of sensory signals.

We evaluated estimated slopes by simulating decisions from known boundaries and comparing estimated values with known values. Decisions were simulated using two alternative models: (a) a rise-to-threshold model, with noisy accumulation of evidence but no noise in the boundary and (b) a rise-to-threshold model with no noise in the accumulated signal but noisy *wait–go* decisions. Once decisions had been generated, slopes were estimated using two alternative fitting methods: the logistic regression method described in Equations 2 and 3 and a maximum likelihood fit to rise-to-threshold model described below. In Figure B1, we show a 2 × 2 comparison between the two estimation methods and the two models used to generate the decisions. Before discussing these results, we describe how simulated data was generated and how the slopes of boundary were estimated.[Fig-anchor fig13]

### Simulating Decisions

In the noisy accumulation model, each sample (cue) was generated using a Bernoulli process (Equation 1), with drift parameters (*u*_*e*_ = 0.72, *u*_*d*_ = 0.50). These samples were then integrated in a noisy decision variable, *V*:

$$V_{t+1}=V_{t}+\delta X+\xi$$ 5

where $\xi ∼N(\cdot |0,\;\sigma_{V}^{2})$ is a zero-mean Gaussian noise with variance σ_*V*_^2^. This integration process generates a random walk, (*X*_1_, . . . , *X*_*t*_) and terminates when the decision-variable, *V*_*t*_, crosses a known boundary. We simulated random walks using 200 different values of boundary (standing for 200 simulated participants), with varying slopes and intercepts, generating 1,000 random walks for each boundary (in both easy and mixed conditions).

For the stochastic boundary model, decisions were generated stochastically based on the distance from boundary. For each given boundary, θ, we determined the probability of going at any point (*t*, *x*) of the random walk, based on the distance, *f*_θ_(*t*, *x*), of the point from the boundary:

$$P(A=go)=\frac{e^{f_{\theta }(t,x)}}{1+e^{f_{\theta }(t,x)}}$$ 6

The key difference between data simulated using this model and the rise-to-threshold model is that this model does not assume any accumulation of noise—each *wait–go* action is independent and solely based on P(A=go) at that point.

### Estimating Slopes

The logistic regression method of estimating slopes has been described in the main text (Equations 2 and 3). We now describe how we generated maximum-likelihood estimates of slopes for the rise-to-threshold model with noisy accumulation of evidence.

According to this model, the value of the decision variable after accumulating *t* samples and evidence *x* is obtained by integrating Equation 5 and is given by the Gaussian distribution, $N(\cdot |x,\;\sigma_{d}^{2}t)$, with mean *x* and variance σ_*d*_^2^*t*, where σ_*d*_ is a free parameter that needs to be estimated. The probability of observing a *go* at (*t*, *x*) will be given by the probability that the decision variable is greater than or equal to the boundary, θ, i.e.

$$\begin{array}{ll} P(t^{go},\;x^{go}) & =\;\int_{\theta (t,\;x)}^{\infty }N(\kappa |x,\;\sigma_{d}^{2}t)d\kappa \\ & =\;1-\Phi (f_{\theta }(t,\;x)|x,\;\sigma_{d}^{2}t) \end{array}$$ 7

where *f*_θ_(*t*, *x*) is the distance to the current evidence, *x*, from the boundary and Φ(·|*x*, σ_*d*_^2^*t*) is the cumulative Gaussian with mean *x* and variance σ_*d*_^2^*t*. The boundary θ is parameterised by it’s slope, *m*, and intercept, *c*. Both of these are free parameters of the model. Similarly, the probability of *wait*ing at (*t*,*x*) is given by Φ(*f*_θ(*t*, *x*)_|*x*, σ_*d*_^2^*t*). If $D^{wait}$ is the set of all wait observations, {(*t*_1_^*wait*^, *x*_1_^*wait*^), . . . , (*t_n_^wait^*, *x_n_^wait^*)}, and $D^{go}$ is the set of all go observations, {(*t*_1_^*go*^, *x*_1_^*go*^), . . . , (*t_m_^go^*, *x_m_^go^*)}, then the likelihood of all observations is given by

$$L(D|m,\;c,\;\sigma_{d})=\underset{(t,\;x)\in D^{go}}{\prod }\left(1-\Phi (f_{\theta }(t,\;x\right)|x,\;\sigma_{d}^{2}t))\times \underset{(t,\;x)\in D^{wait}}{\prod }\Phi (f_{\theta }(t,\;x)|x,\;\sigma_{d}^{2}t)$$ 8

where D includes both $D^{wait}$ and $D^{go}$ decisions. We obtained estimates, *m̂*, *ĉ* and σ^d that maximized the likelihood function given in Equation 8.^8^

### Evaluation of Estimation Methods

The panel on the top-left of Figure B1 compares the true and estimated difference in slopes when decisions were generated using the noisy accumulation model (Equation 5) and estimated using the logistic regression model (Equations 2 and 3). The estimated difference in slopes is not equal to the true difference and the deviation from truth does depend on the noise in the accumulation process, σ_*V*_; simulations showed that the difference approaches zero as σ_*V*_ approaches zero. Moreover, the estimated difference is approximately proportional to the true difference, so that if statistical test, such as the t-test, is valid on the true difference it will also be valid on the estimated difference.

The top-right panel in Figure B1 compares the true and estimated differences in slopes, when decision were again generated using the noisy accumulation model (Equation 5) and also estimated assuming a noisy accumulation to boundary (Equation 8). It can be seen that the difference in slopes estimated using this method is fairly similar to that estimated using the logistic regression model. The estimated difference is approximately proportional to the true difference and, like the estimates in top-left panel, these estimates also contains a deviation that depends on the diffusion parameter used to generate the data, σ_*V*_. Simulations also showed (not shown in Figure B1) that, while the difference in slopes using this method was similar to that using the logistic regression method, this method overestimated the slopes when true slope was zero—that is, when bounds are in fact flat, it estimates them to be increasing, which is not the case with the logistic regression method used in this study.

The bottom-left panel shows that the difference in slopes estimated using the logistic regression model (Equations 2 and 3) are highly correlated with the true differences when data is simulated using the stochastic boundary model (Equation 6). This is not surprising since this method of generating the data inverts the logistic regression model. In contrast, the bottom-right panel shows that when slopes are estimated using the noisy accumulation process (Equation 8), the correlation between the true and estimated difference in slopes decreases and there is a bias in the estimated difference albeit, again, multiplicative.

To sum up, difference in slopes estimated using the logistic regression model, were at least as good as estimates using the maximum likelihood method and the logistic regression model was, in fact, robust to misspecification of the model that generates the data. Furthermore, estimated differences were linearly related to true differences, which meant that it was valid to use the t-test on the estimated differences for inferring whether there was a difference in the true slopes of single- and mixed-difficulty games.

## C Variable Drift Rate and Information Loss

We have analyzed the data based on the evidence and time of each wait and go decision, assuming that participants accumulated every cue provided to them and the internal drift rate was the same in every trial. In this appendix, we verified whether our inferences about difference in slopes would be valid if some evidence was lost during accumulation and the drift rate varied from trial-to-trial.

Consider a variable drift rate first. Even though our experiments use the expanded-judgement paradigm where the drift of the stimulus is controlled by the experimenter, it is possible that the internal drift rate varies from trial-to-trial due to fluctuations in attention and cognitive resources. Fluctuations in the effect of stimulation have been modeled as a random variable since Thurstone’s comparative and categorical judgement models (Thurstone, 1927a, 1927b) and form an integral part of signal detection theory (Tanner & Swets, 1954; Green & Swets, 1966) and sequential sampling models (Ratcliff, 1978; Ratcliff & Smith, 2004).

To check how variability in drift rate affected our results, we simulated decisions using a rise-to-threshold model both with and without variability in drift and estimated the slopes in each case using the logistic regression method (Equation 3). A comparison between the estimated slopes showed us how a variability in drift affects our estimate.

Decisions were simulated using the following method. Stimuli were generated using a Bernoulli process (Equation 1), with drift ϵ = ϵ_0_ + ν, where ϵ_0_ was a constant drift parameter based on the type of game (e.g., ϵ_0_ = 0.22 for easy games) and $\nu ∼N(0,\;\sigma_{drift}^{2})$ was a random variable drawn independently for every trial. The overall drift, ϵ, was bound between 0 and $\frac{1}{2}$. These stimuli were then integrated in a noisy decision-variable, *V*:

$$V_{t+1}=V_{t}+\delta X+\xi$$ 9

where $\xi ∼N(0,\;\sigma_{V}^{2})$ is a zero-mean Gaussian noise with variance σ_*V*_^2^. This integration process generated a random walk, (*X*_1_, . . . , *X*_t_) and terminated when the decision variable, *V*_*t*_, crossed a known boundary. We simulated random walks using 200 different values of boundary (standing for 200 simulated participants), with varying slopes and intercepts, generating 1,000 random walks for each boundary (in both easy and mixed conditions).

Figure C1 (Panel a) shows a comparison between true and estimated slopes in easy games when σ_*drift*_ = 0, i.e. there was no trial-to-trial variability in drift, as well as when σ_*drift*_ = 0.35, that is, there was a large trial-to-trial drift variability. When there was no trial-to-trial variability in drift, the estimated slopes were close to true slopes. In the presence of drift variability, the magnitude of slopes was systematically overestimated. However, constant slopes were still estimated as constant and increasing or decreasing slopes were also estimated as increasing or decreasing, respectively. Thus, irrespective of trial-to-trial variability in drift rates, a negative estimate of slope indicated that the true slope was also negative.[Fig-anchor fig14]

Figure C1(b) shows a comparison between true and estimated difference in slopes for σ_*drift*_ = 0 and σ_*drift*_ = 0.35. In the presence of drift variability, the bias in estimation of slopes results in a bias in estimation of difference in slopes, with the magnitude of estimated difference being larger than true difference. The estimated difference in slopes is approximately proportional to the true difference for both σ_*drift*_ = 0 and σ_*drift*_ = 0.35. Therefore, when there was no difference in true slopes, there was also no difference in estimated slopes. Similarly, when true difference in slopes was positive (negative), the estimated difference in slopes was also positive (negative). Therefore, even when there was trial-to-trial variability in drift, the estimated difference in slopes indicated a difference in true slopes.

We also checked whether our inferences were robust to loss of information in the integrative decision process. To do this, we simulated data from the binomial loss model (Smith & Vickers, 1989), where observations δ*X* available to the decision maker are only accumulated with some fixed probability *p*_*a*_, otherwise they are lost:

$$V_{t+1}=\{\begin{array}{ccc} V_{t}+\delta X+\xi & \text{with probability} & p_{a}\\ V_{t}+\xi & \text{with probability} & 1-p_{a} \end{array}$$ 10

where *V_t_* and ξ are defined in the same way as Equation 9. Figure C2(a) compares the estimates of slopes during easy games for 200 simulated participants from the information loss model (Equation 10, *p_a_* = 0.7) with 200 simulated participants from the rise-to-threshold model without any information loss (Equation 9). Introducing information loss resulted in some systematic biases: estimated slopes were, in general, larger and shifted in the positive direction so that constant slopes were estimated to have a small positive value and slightly negative slopes were estimated to be constant, whereas large negative and positive slopes were estimated to have a larger value than the true slopes. Note the direction of this bias—when slopes were estimated to be negative (as in the majority of experiments in this study), the true slopes were also negative while when they were estimated to be positive, they could in fact be constant. This makes sense: the total information loss will increase over time and since the drift is positive, decisions made at later points of time will seem to be at higher levels of evidence than internally integrated.

Figure C2(b) compares the true and estimated difference in slopes between easy and mixed games. When information was lost, the estimate of slopes in each type of game was biased, therefore, the estimate for difference in slopes was also biased. However, the estimated difference in slopes was still approximately proportional to true difference in slopes, passing through the origin so that the estimated difference in slopes was proportional to the true difference in slopes even when data were simulated from the binomial loss model.[Fig-anchor fig15]

## D Model Selection and Recovery

A key finding of Experiments 1 and 2 is that participants seem to decrease their decision boundaries with time, especially in the mixed-difficulty condition. To check the robustness of this result, we compared the logistic regression model presented in the main text (Equation 2), which uses both evidence and time to predict the probability of *go*, with a simpler model that uses only evidence to predict this probability:

$$\log\frac{P(A=go)}{P(A=wait)}=\beta_{0}+\beta_{X}*X$$ 11

where β_*X*_ is the regression coefficient for evidence and β_0_ is the intercept. Note that this simpler model is equivalent to assuming that decision boundaries do not change with time as only evidence predicts whether a participant chooses the action wait or go during a trial. We inferred the preferred model for each participant and condition by computing the Bayesian information criterion (BIC) for each model (Schwarz, 1978; Wagenmakers, 2007). Following Wasserman (2000) and Hawkins et al. (2015), we approximated the posterior probability for a participant using each model under the assumption that both models are a priori equally likely,

$$P_{BIC}(M_{i}|Data)=\frac{exp(-\frac{1}{2}BIC(M_{i}))}{\sum_{j=1}^{m}exp(-\frac{1}{2}BIC(M_{j}))}$$ 12

Figure D1 plots these posterior probabilities for data from both single-difficulty (left column) and mixed-difficulty conditions (right column) for each participant. Shaded (red) bars show the posterior model probability for the more complex model (using both evidence and time), while the while hatched bars show the complementary posterior probability for the simpler model (only on evidence).[Fig-anchor fig16]

In agreement with the slopes estimated using the line of indifference (Figures 5 and 8), we found that the model using both evidence and time provided the best account of the data in Experiments 1, 2a, and 2b. This was especially true for the mixed-difficulty games. Note that, in spite of the fact that the BIC rewards lower model complexity, the simpler model of Equation 11 provided the best account for only 5 participants during the mixed-difficulty trials during these experiments. Data from most other participants was better accounted for by the model using time as an additional predictor variable.

In contrast, during Experiments 2c and 2d, where the inter-trial intervals were same for correct and incorrect decisions and reward landscapes were flatter (Figure 9), support for the two models was much more mixed. Finally, during Experiment 3, where the reward landscape favored constant (or slightly increasing) decision boundaries with time (Figures 10 and 11), the simpler model, using only evidence to predict probability of going, provided the best account of the data, especially in the mixed-difficulty condition.

We checked the validity of this model selection procedure using a model recovery analysis. We simulated decisions using two noisy accumulation-to-threshold models (see Equation 5 in Appendix B). The first model used a constant threshold, while the second one used a threshold that decreased linearly with time. For each simulated participant, we generated 100 random walks with drifts drawn, with uniform probability, from the set ϵ ∈ {0.22, 0}. Thus, these simulated random walks approximately matched the data collected and conditions for mixed-difficulty trials in Experiment 2.

We then fit the two logistic regression models discussed above, one using only evidence as a predictor (Equation 11) and the other using evidence as well as time (Equation 2), to each of these simulated participants and computed the BIC values for each fit. Each plot in Figure D2 shows the distribution of difference in BIC values for 400 simulated participants, 200 of which are simulated using the fixed boundary model (hatched distribution), whereas the remaining 200 are simulated using the decreasing boundary model (shaded distribution). It can be seen from these plots that the decisions generated using a fixed boundary model are better fit (lower BIC_*fix*_) by the logistic regression model using only evidence as the predictor, while the decisions generated using a decreasing boundary model are better fit (lower BIC_*var*_) by the logistic regression model using both evidence as time as predictors. Furthermore, when the slope of the decision boundaries is increased for the simulated decisions, the difference in BIC values increases (compare the shaded region in the three plots), showing that the BIC of the time-varying bounds model decreases with increase in the slope used to generate the decisions.[Fig-anchor fig17]
